# Early IL-6 signalling promotes IL-27 dependent maturation of regulatory T cells in the lungs and resolution of viral immunopathology

**DOI:** 10.1371/journal.ppat.1006640

**Published:** 2017-09-27

**Authors:** Chloe J. Pyle, Faith I. Uwadiae, David P. Swieboda, James A. Harker

**Affiliations:** 1 Section of Inflammation, Repair and Development, National Heart and Lung Institute, Imperial College London, South Kensington Campus, South Kensington, London, United Kingdom; 2 MRC & Asthma UK Centre in Allergic Mechanisms for Asthma, London, United Kingdom; University of Pennsylvania, UNITED STATES

## Abstract

Interleukin-6 is a pleiotropic, pro-inflammatory cytokine that can promote both innate and adaptive immune responses. In humans with respiratory virus infections, such as Respiratory Syncytial Virus (RSV), elevated concentrations of IL-6 are associated with more severe disease. In contrast the polymorphisms in the *Il6* promoter which favour lower IL-6 production are associated with increased risk of both RSV and Rhinovirus infections. To determine the precise contribution of IL-6 to protection and pathology we used murine models of respiratory virus infection. RSV infection resulted in increased IL-6 production both in the airways and systemically which remained heightened for at least 2 weeks. IL-6 depletion early, but not late, during RSV or Influenza A virus infection resulted in significantly increased disease associated with an influx of virus specific T_H_1 and cytotoxic CD8^+^ T cells, whilst not affecting viral clearance. IL-6 acted by driving production of the immunoregulatory cytokine IL-27 by macrophages and monocytes, which in turn promoted the local maturation of regulatory T cells. Concordantly IL-27 was necessary to regulate T_H_1 responses in the lungs, and sufficient to limit RSV induced disease. Overall we found that during respiratory virus infection the prototypic inflammatory cytokine IL-6 is a critical anti-inflammatory regulator of viral induced immunopathology in the respiratory tract through its induction of IL-27.

## Author summary

In clearing a respiratory virus, the host must strike a careful balance between the need to clear the infection and the potential of the immune response to damage the delicate structure of the lungs. Here we show that Interleukin-6, a soluble mediator commonly associated with inflammation and seen in humans with severe respiratory infection, is actually critical in promoting the resolution of the host response to respiratory virus infection and limiting disease. We have found that the early production of IL-6 after infection promotes the production of the regulatory mediator Interleukin-27 by lung resident immune cells, which in turn drives suppression of otherwise damaging inflammation. Removal of either IL-6 or IL-27 enhances disease during viral infection, while restoration of IL-27 is sufficient to allow faster recovery. Thus we have identified a novel immunological network within the respiratory tract which accelerates recovery after respiratory virus infection.

## Introduction

The prototypic pro-inflammatory cytokine interleukin 6 (IL-6) is upregulated in a wide variety of immunological conditions including autoimmunity, cancer, infection and vaccination (reviewed in [1]). IL-6 signalling influences both innate and adaptive immunity by regulating a broad range of processes including cell proliferation, differentiation, survival and inflammation. For instance IL-6 controls the accumulation of neutrophils at the site of inflammation [2, 3] and is known to influence macrophage differentiation [4–6]. In addition IL-6 promotes CD4^+^ and CD8^+^ T cell survival, and there is extensive literature regarding IL-6’s ability to drive the differentiation of naïve CD4^+^ T cells into T helper 17 (T_H_17) cells while suppressing regulatory T cell (Treg) development and function *in vitro* [7]. It is also known to promote the differentiation of germinal centre (GC) B cells and T follicular helper cells (T_FH_), and is therefore critical for the production of high affinity antibody [8–10].

Fitting with IL-6’s pleiotropic roles in immunity it is often produced early after infection, but has distinct effects on the outcome depending on the type of infection. IL-6 deficient mice are more susceptible to bacterial infection with *Listeria monocytogenes* or H5N1 Influenza A virus (IAV) due to neutrophil defects [11, 12]. During Vaccinia virus or IAV H1N1 infection IL-6 promotes anti-viral CD8^+^ T cell responses to enhance viral clearance [13, 14]. After infection with vesicular stomatitis virus or a chronic variant of lymphocytic choriomeningitis virus (LCMV) IL-6 promotes T-dependent antibody responses [8, 13]. In contrast IL-6 is not required for control of the acute variant, LCMV Armstrong 53b, and its absence does not appear to affect either cytotoxic T cell or antibody mediated immunity [8, 13].

Respiratory syncytial virus (RSV) is a negative strand RNA virus of the *Pneumoviridae* family. It is a major cause of lower respiratory tract illness and the most common cause of infant hospitalization in the western world (reviewed in [15]). RSV infection is associated with elevated concentrations of a broad spectrum of cytokines and chemokines, including IL-6, both in airways of humans and experimentally infected mice [16, 17]. The nature of the cytokine response is crucial in determining the outcome of RSV infection. For instance polymorphisms in the type I interferon (IFN) pathway are associated with enhanced susceptibility to RSV bronchiolitis in infants [18], and type I IFN receptor deficiency results in reduced viral clearance and enhanced disease severity in mice [19]. Likewise, deletion or depletion of IL-10 results in enhanced T cell mediated immunopathology and more severe disease [20–22]. IL-6 can be rapidly secreted by both bronchial epithelial cells and alveolar macrophages upon exposure to RSV [23, 24] and *in vivo* the presence of alveolar macrophages is essential for the production of IL-6 and other inflammatory mediators including type I IFNs and TNF [25]. Importantly heightened IL-6 concentrations are found in infants hospitalized with RSV [26], but paradoxically a single nucleotide polymorphism in the *Il6* promoter at position 174, the 174-C/C genotype, is associated with a low IL-6 production phenotype and greater illness upon natural RSV infection [27–29].

We used IL-6 depletion during experimental RSV infection of mice to delineate the role of IL-6 signalling in disease. IL-6 was rapidly produced in the airways and lung tissue of mice after RSV infection and remained detectable until after disease resolution. Depletion of IL-6 in the early stages of infection resulted in enhanced disease characterized by an influx of IFN-γ secreting virus specific T cells into the lungs and airways, dysregulation of regulatory T cell responses and reduced production of the immune-regulatory cytokines IL-10 and IL-27. Depletion of IL-27 in the airways during RSV infection mirrored enhanced disease seen after IL-6 depletion and local administration of IL-27 to the airways was sufficient to prevent the enhanced disease seen in the absence of IL-6 and restore regulatory T cell maturation. Thus, IL-6 prevents enhanced pathology after respiratory tract infection by promoting local IL-27 induced immune regulation.

## Results

### IL-6 is produced rapidly after RSV infection and is critical for resolution of disease

**Fig 1:**
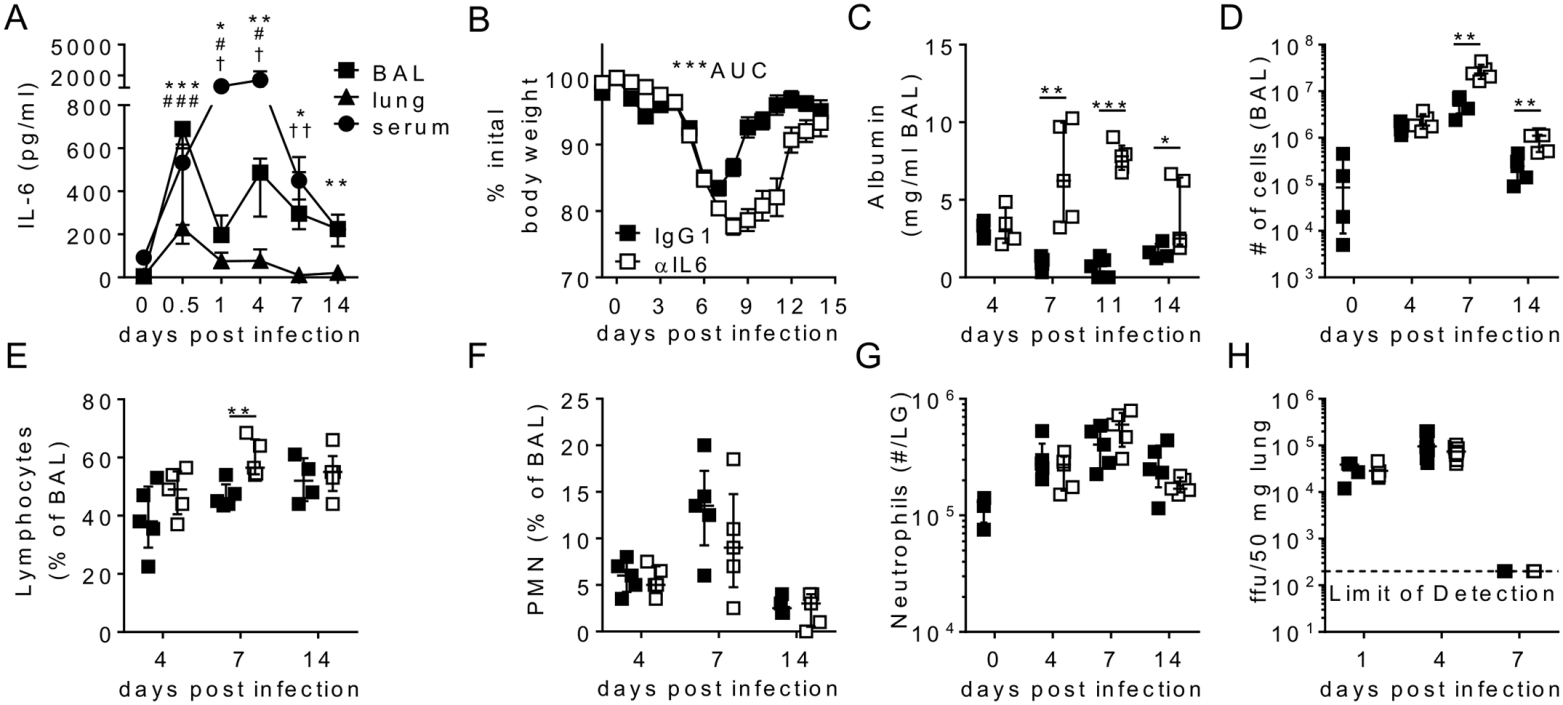
IL-6 promotes resolution of RSV-mediated disease. 8 week old BALB/c mice were infected with 8 x 10^5^ ffu of RSV A2 i.n. (A) IL-6 was measured by ELISA in the BAL, lung tissue and serum. (B-H) RSV infected mice were given 0.5 mg of either HRPN (IgG_1_) or MP5-20F3 (αIL-6) i.p. on day -1 p.i. and 0.25 mg i.p. every other day after that. (B) The percentage of weight on day 0 was measured daily, area under the curve (AUC) was used to test statistical significance, (C) Albumin was measured in the BAL by ELISA. (D) The number of BAL cells was counted and (E) lymphocytes and (F) Neutrophil (PMN) frequencies were determined by H&E staining. (G) Lung neutrophils (defined as Ly6G^+^CD11b^+^CD90^-^CD19^-^autofluorescence^-^) were enumerated by flow cytometry. (H) Viral load in the lungs was determined by focus forming assay. For (A) * represents BAL, ^#^ represents lung and ^†^ represents serum. Data is representative of n = 2 independent repeats of n = 5 mice per time point except B which represents 20 mice per group.

To determine whether IL-6 played a role in viral control and disease during RSV infection we first assessed the timing and location of its production. Intranasal infection of adult BALB/c mice with RSV resulted in significantly increased concentrations of IL-6 by 12 hours post infection (p.i.) in the airways (bronchoalveolar lavage (BAL)) (Fig 1A). IL-6 concentrations reduced after this time point but remained significantly elevated in the airways up to 14 days p.i. compared to uninfected mice. IL-6 concentrations in the lung followed a similar pattern to those observed in the airways (Fig 1A). Significantly increased amounts of IL-6 were also detectable in the serum, although here the highest concentrations were seen at day 4 p.i. (Fig 1A). Together this data showed that IL-6 was produced rapidly upon RSV infection, both locally and systemically, and remained detectable throughout the course of infection.

To determine the role of this IL-6 we next treated mice with a neutralizing anti-IL-6 antibody 1 day prior to RSV infection, with further doses administered every other day throughout the course of the experiment, which was sufficient to deplete IL-6 in the airways, lungs and serum (S1 Fig). Primary RSV infection of adult BALB/c mice causes a progressive weight loss, which peaks between 6 and 8 days post infection and is associated with lymphocyte influx into the lungs and airways [25]. In the absence of IL-6, peak weight loss was greatly increased, and a return to normal body weight was delayed from day 10 p.i. until day 14 p.i. compared to the isotype control (Fig 1B). In addition 5 out of 20 anti-IL-6 treated mice had to be euthanized at day 11 p.i. as they had reached the study’s humane endpoint, while all isotype treated mice showed complete recovery from infection. IL-6 depletion also resulted in elevated airway albumin, a measure of vascular permeability and damage in the lungs, at days 7, 11 and 14 p.i. compared to IgG_1_ treated mice (Fig 1C). After IL-6 neutralization mice had significantly heightened airway cell counts at both days 7 and 14 p.i. compared to control mice (Fig 1D). Cellular infiltration into the airways was largely lymphocytic, with IL-6 depleted mice having increased lymphocyte proportions at day 7 p.i., compared to IgG_1_ treated mice, while the proportion of airway neutrophils present was similar irrelevant of treatment (Fig 1E & 1F). IL-6 has been shown to be essential for promoting neutrophil dependent viral control after influenza A virus infection, by promoting neutrophil survival [12], however there was no significant difference in lung neutrophil numbers between anti-IL6 and isotype control treated mice after RSV infection (Fig 1G). Importantly both early, 24 hours p.i., and peak, 4 days p.i, lung viral loads were similar between IL-6 depleted and control mice (Fig 1H) and all mice also successfully cleared the virus from their lungs by day 7 p.i., irrespective of treatment.

Overall this showed that IL-6 was critical for regulating disease severity during RSV infection, with loss of IL-6 correlating to increased weight loss, airway cell infiltration by a range of immune cells and vascular leakage into the airway. Importantly, however, this effect seemed independent of the outcome of the infection, as IL-6 was not necessary for control or clearance of RSV.

### IL-6 dampens RSV-specific T cell responses

**Fig 2:**
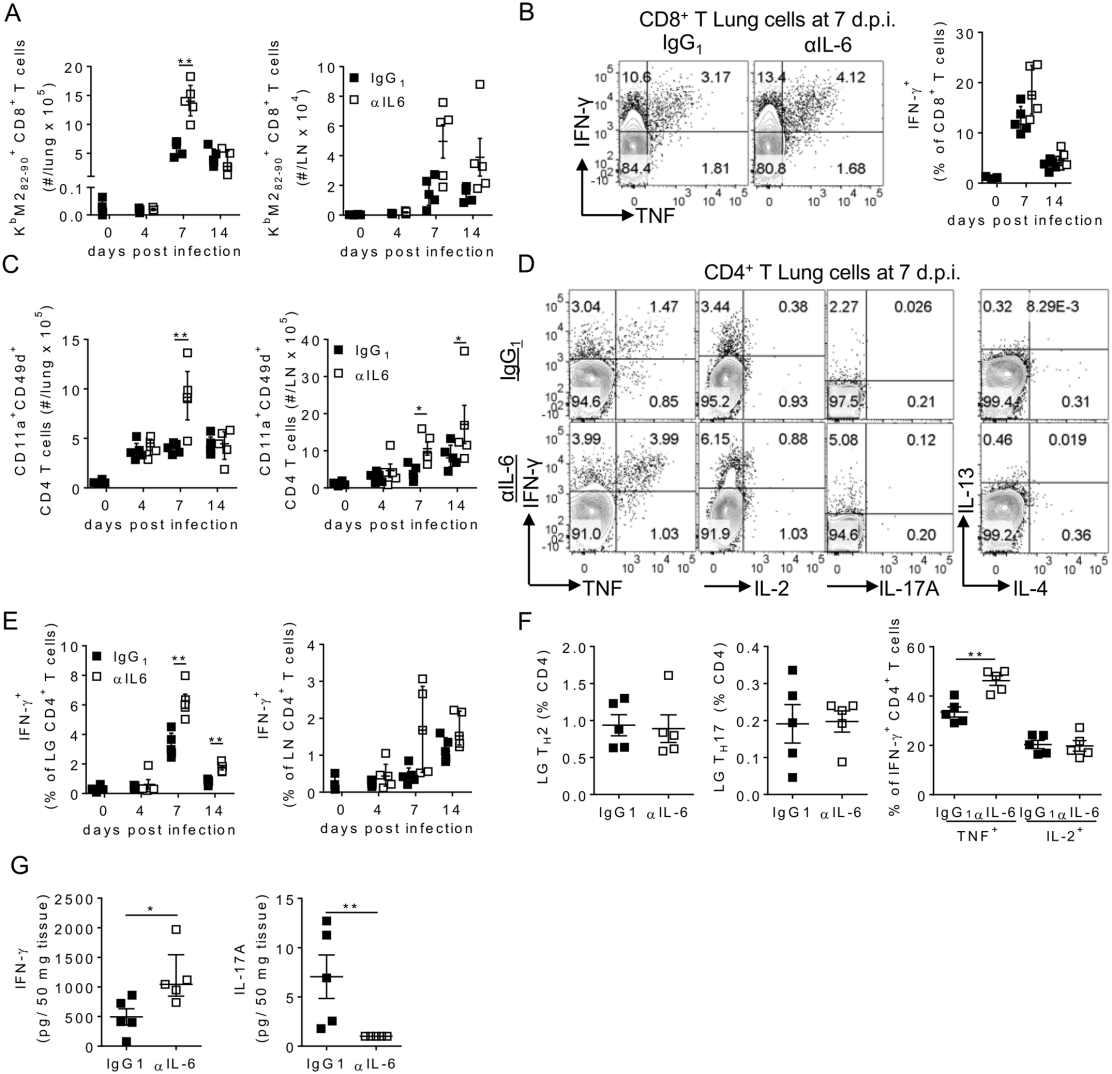
IL-6 depletion results in enhanced virus specific T cell responses. 8 week old BALB/c mice were infected with 8 x 10^5^ ffu of RSV A2 i.n. and given 0.5 mg of either HRPN (IgG_1_) or MP5-20F3 (αIL-6) i.p. on day -1 p.i. and 0.25 mg i.p. every other day after that. Mice were euthanized at days 4, 7 and 14 p.i. Flow cytometry was used to determine (A) the number of K^b^M2_82-90_^+^ CD8^+^ T cells, (B) the proportion of IFN-γ^+^ and TNF^+^ lung CD8^+^ T cells following M2_82-90_ stimulation, (C) the number of CD11a^+^CD49d^+^ CD4^+^ T cells and (D-F) the proportion of IFN-γ_+_ at different days p.i., and TNF^+^, IL-2^+^, IL-17^+^, IL-13^+^ and IL-4^+^ CD4^+^ T cells at day 7 p.i. following stimulation with RSV F_51-66_, P_39-55_ and G_181-197_ peptides *ex vivo* in the lungs and lymph nodes. (G) IFN-γ and IL-17 were measured in lung tissue at day 7 p.i. by ELISA. Representative FACS plots are lungs at day 7 p.i.. Data is representative of n = 2 independent repeats of n = 5 mice per time point.

Immunopathology, rather than viral replication, is known to be the critical driver of disease severity during primary RSV infection of mice. Indeed weight loss strongly correlates with the frequency of virus specific CD8^+^ T cells in the lungs, and depletion of CD8^+^ T cells during RSV infection is sufficient to prevent weight loss [25, 30]. In our studies the number of RSV specific K^b^M2_82-90_^+^ immunodominant CD8^+^ T cells peaked between days 7 and 14 p.i. in RSV infection of WT mice. IL-6 depletion resulted in significantly increased proportions and numbers of K^b^M2_82-90_^+^ CD8^+^ T cells in the lungs at day 7 p.i. and mediastinal lymph nodes at days 7 and 14 p.i. (Fig 2A and S2 Fig), although the proportion of CD8^+^ T cells secreting IFN-γ in response to M2_82-90_ stimulation was similar (Fig 2B). The frequency of CD11a^+^ CD49d^+^ CD4^+^ T cells has been used to measure polyclonal antigen specific CD4^+^ T cell responses in a variety of infections including RSV [31–33]. After RSV infection CD11a^+^ CD49d^+^ T cell numbers rapidly increased in both the lungs and mediastinal lymph nodes of mice (Fig 2C). As with the CD8^+^ T cell response, IL-6 depletion resulted in enhanced lung CD4^+^ T cell responses at day 7 p.i. and enhanced lymph node CD4^+^ T cell responses at days 7 and 14 p.i. (Fig 2C). RSV specific stimulation (using RSV F_51-66_, P_39-55_ and G_181-197_ peptides [34]) predominantly resulted in IFN-γ^+^ CD4^+^ T cells, the frequency of which was significantly increased in the absence of IL-6 (Fig 2D & 2E). Little or no IL-17A^+^, IL-13^+^ or IL-4^+^ T cell were detected after RSV infection, irrespective of IL-6 depletion (Fig 2D & 2F). IL-6 depletion did however result in increased TNF secretion by virus specific IFN-γ^+^ CD4^+^ T cells (Fig 2F). IFN-γ concentrations in the lungs at day 7 p.i. were significantly elevated in the absence of IL-6, supporting the presence of increased IFN-γ secreting cells (Fig 2G). In contrast a low, but detectable, concentration of IL-17A was present in isotype treated mice, which was absent in αIL-6 treated mice (Fig 2G). Overall, depletion of IL-6 enhanced the number and function of virus specific T_H_1 and CD8^+^ T cells and at the peak of disease severity, without significantly altering either T_H_2 or T_H_17 responses.

### IL-6 regulates the maturation of lung regulatory CD4^+^ T cells

**Fig 3:**
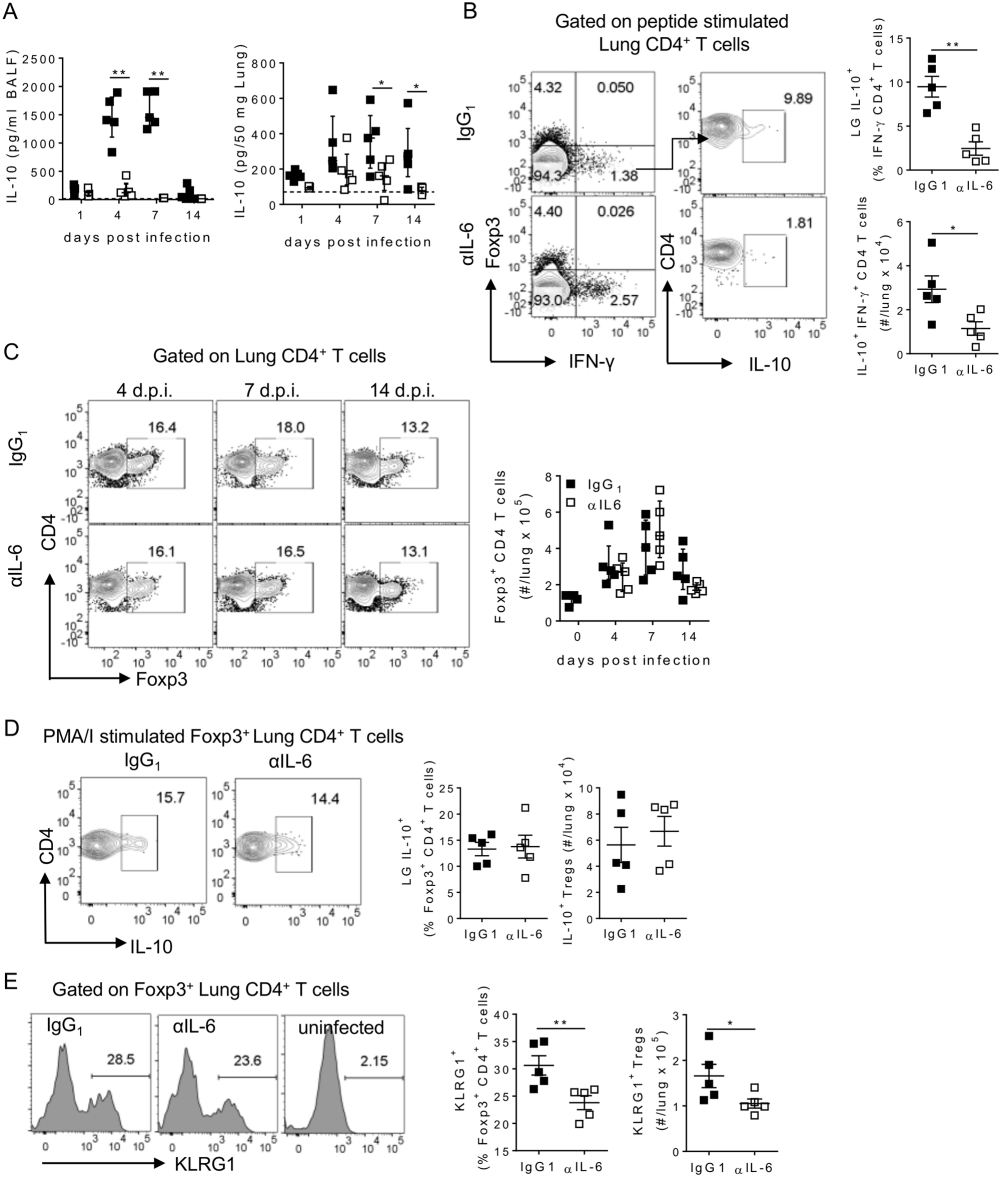
IL-6 promotes the maturation of regulatory CD4^+^ T cells in the lungs. 8 week old BALB/c mice were infected with 8 x 10^5^ ffu of RSV A2 i.n. and given 0.5 mg of either HRPN (IgG_1_) or MP5-20F3 (αIL-6) i.p. on day -1 p.i. and 0.25 mg i.p. every other day after that. (A) IL-10 was measured in the airways and lungs at multiple timepoints post infection by ELISA. (B-E) At day 7 p.i. (B) The frequency of IL-10^+^ in lung IFN-γ^+^ CD4^+^ T cells at day 7 p.i. was determined after RSV F_51-66_, P_39-55_ and G_181-197_ peptide stimulation. (C) The frequency of lung Foxp3^+^ CD4^+^ T cells. (D) The proportion and number of IL-10^+^ after PMA and ionomycin stimulation and (E) KLRG1^+^ Tregs in the lung. Data is representative of n = 2 independent repeats of n = 5 mice per time point.

The resolution of disease following RSV infection is mediated via multiple pathways, with Foxp3^+^ Tregs and IL-10, derived primarily from Foxp3^-^ secreting type 1 regulatory T (Tr1) cells, particularly important in curbing the virus specific T cell response in the lungs and airways [20–22, 35–37]. IL-6 depletion resulted in significantly reduced IL-10 concentrations in both the airways and lungs at both days 4 and 7 p.i. (Fig 3A). As with other virus specific CD4^+^ T cells, virus specific Tr1 cells (IFN-γ^+^ IL-10^+^ Foxp3^-^) CD4^+^ T cells were not detectable in the lungs of RSV infected mice at day 4 p.i, after *ex vivo* peptide stimulation. However by day 7 p.i. Tr1 cells were detectable in the lungs at low frequencies and the proportion and number of IFN-γ^+^ CD4^+^ T cells that were producing IL-10 was significantly lower after IL-6 depletion than in controls (Fig 3B). The proportion and number of Foxp3^+^ Tregs present in the lungs was not affected by the absence of IL-6, (Fig 3C). Peptide stimulation did not elicit detectable cytokine production from Foxp3^+^ CD4 T cells, concordant with the idea that Tregs, especially those of thymic origin, recognise a distinct repertoire of antigens to their effector cell counterparts [38]. Polyclonal stimulation however indicated that Treg derived IL-10 was not affected by IL-6 depletion (Fig 3D). There was also a small number of IFN-γ^+^ Tregs which were also unaffected by loss of IL-6 and which were largely IL-10^-^. In contrast the frequency of KLRG-1^+^ Tregs, which represent a functionally mature subset of regulatory T cells [39, 40], was significantly decreased in the lungs in the absence of IL-6 (Fig 3E). The reduced frequency of IL-10^+^ Tr1 cells and KLRG1^+^ Tregs after IL-6 depletion may indicate that IL-6 plays a pivotal role in driving resolution of inflammation after RSV infection via promoting regulatory T cell subsets in the lungs.

### IL-6 signalling during peak T cell responses does not influence RSV mediated immunopathology

**Fig 4:**
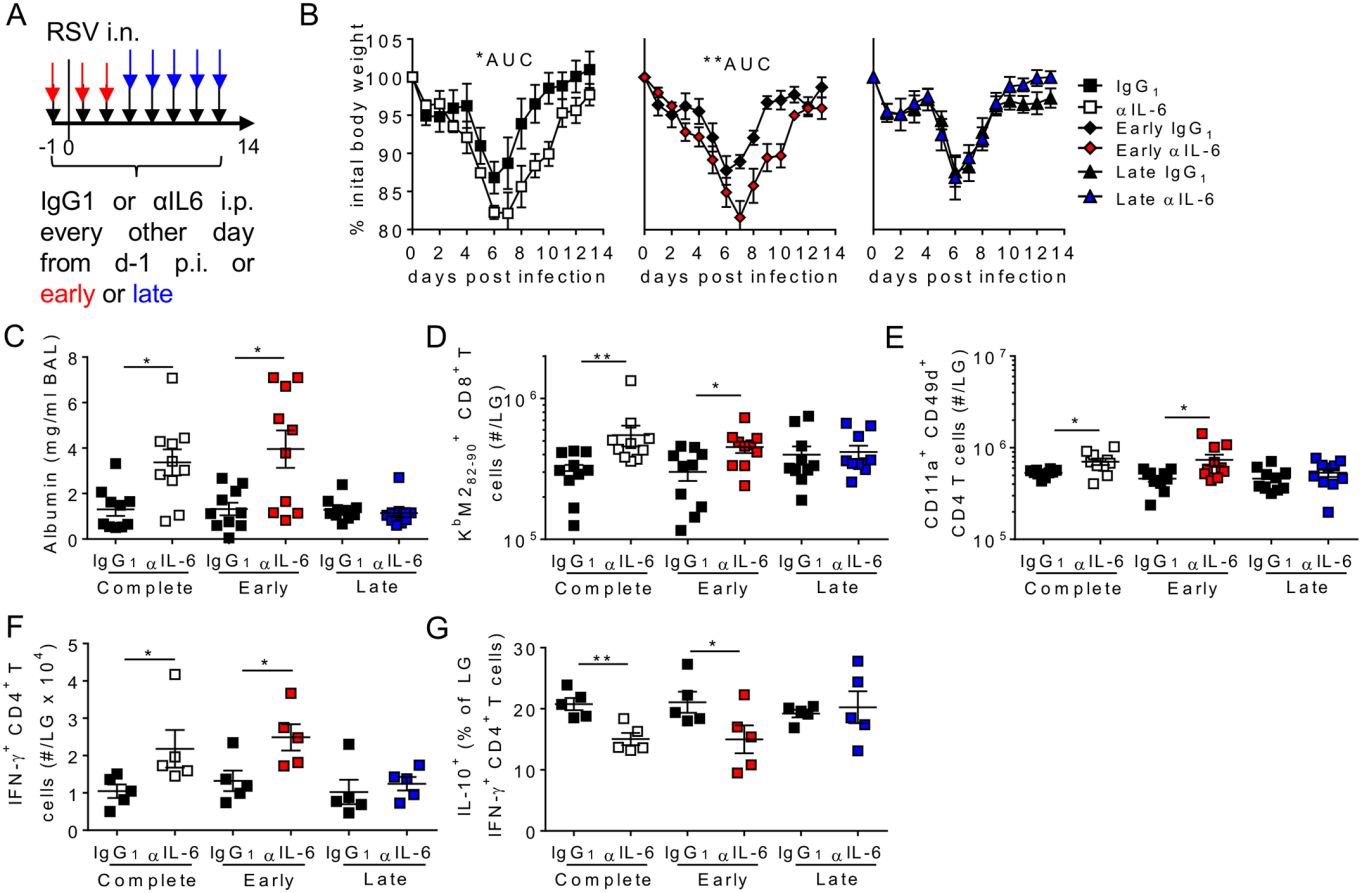
Early, but not late, IL-6 signalling is required for the resolution of RSV induced immunopathology. 8 week old BALB/c female mice were infected with 8 x 10^5^ ffu of RSV A2 i.n. and dosed with either αIL-6 or isotype control antibody as shown in A. (B) Weight loss was monitored daily, area under the curve (AUC) was used to test statistical significance. (C) At day 14 p.i. airway albumin was measured by ELISA. (D-G) At the same timepoint virus specific CD8^+^ T cells (D), antigen experienced CD4^+^ T cells (E), virus specific IFN-γ^+^ CD4^+^ T cells (F) and the proportion of those cells that were IL-10^+^ (G) were determined in the lungs by flow cytometry. A-E represent n = 10 mice per group from 2 independent experiments. F and G are n = 5 mice per group and are representative of 2 independent experiments.

Virus specific CD4^+^ T cells were present at low frequencies for the first 4–5 days post infection before undergoing rapid clonal expansion and recruitment with peak numbers seen in the lungs at between 7 and 14 days post RSV infection. IL-6 meanwhile is produced rapidly upon initial RSV infection with peak concentrations seen within the first 24 hours, although it remains detectable in the airways for several weeks post infection. We therefore sought to determine when IL-6 signalling was required for the resolution of RSV specific T cell responses and disease. IL-6 neutralization antibodies were administered either as before (throughout infection), or ‘early’, from days -1 to 3 p.i., or ‘late’, from days 5 to 13 p.i. (Schematic shown in Fig 4A).

Depletion of IL-6 early, but not late, resulted in exacerbated weight loss and increased clinical symptom scores similar to those seen when IL-6 was depleted throughout (Fig 4B & S3 Fig). Airway albumin was also elevated in mice given early αIL-6 compared to their control group at day 14 p.i. (Fig 4C). In addition mice receiving early αIL-6, but not late αIL-6, treatment had significantly increased virus specific CD8^+^ T cells, antigen specific CD4^+^ T cells and virus specific IFN-γ^+^ CD4^+^ T cells in the lungs compared to isotype control treated mice (Fig 4D–4F). IL-10 secretion by IFN-γ^+^ CD4^+^ T cells present in the lungs was also lower in early, but not late, αIL-6 treated mice (Fig 4G).

Similar results were obtained when mice treated between days -1 and 3 p.i. with αIL-6 were infected with mouse adapted H1N1 IAV PR8 (strain A/Puerto Rico/8/1934 H1N1), with IL-6 depleted mice showing delayed recovery from infection and reduced IL-6, IL-10 and IL-27 in the airways at day 10 p.i. (S4A and S4B Fig). Concomitantly they had increased IFN-γ levels and enhanced T cell infiltration, especially IFN-γ^+^ CD4 T cells, in the lungs (S4C–S4F Fig). IL-10 production by the IFN-γ^+^ CD4 T cells was similar, as were Treg numbers, however the frequency of KLRG1^+^ and IL-10^+^ Tregs were significantly reduced in the absence of IL-6 (S4F–S4H Fig). Lung neutrophil numbers were not affected by the depletion of IL-6 (S4I Fig).

The failure of IL-6 depletion to directly influence disease severity or T cell responses at the peak of inflammation suggests that IL-6 may not be directly acting on virus specific or regulatory T cells. In contrast early IL-6 was essential in regulating the immune response to RSV and IAV, indicating that early IL-6 signalling may promote later immune-regulatory events.

### IL-6 promotes myeloid derived IL-27 during RSV infection

Given the kinetics of IL-6 production and its involvement in regulating disease it seemed likely that IL-6 was acting indirectly to regulate T cell responses. IL-27 is a major inducer of IL-10 production by Tr1 cells (reviewed in [41]) and can also promote the functional maturation of Tregs [42, 43]. Therefore we next analysed IL-27 responses upon RSV infection and investigated whether IL-27 signalling could underlie the observed effects of IL-6 on disease resolution.

**Fig 5:**
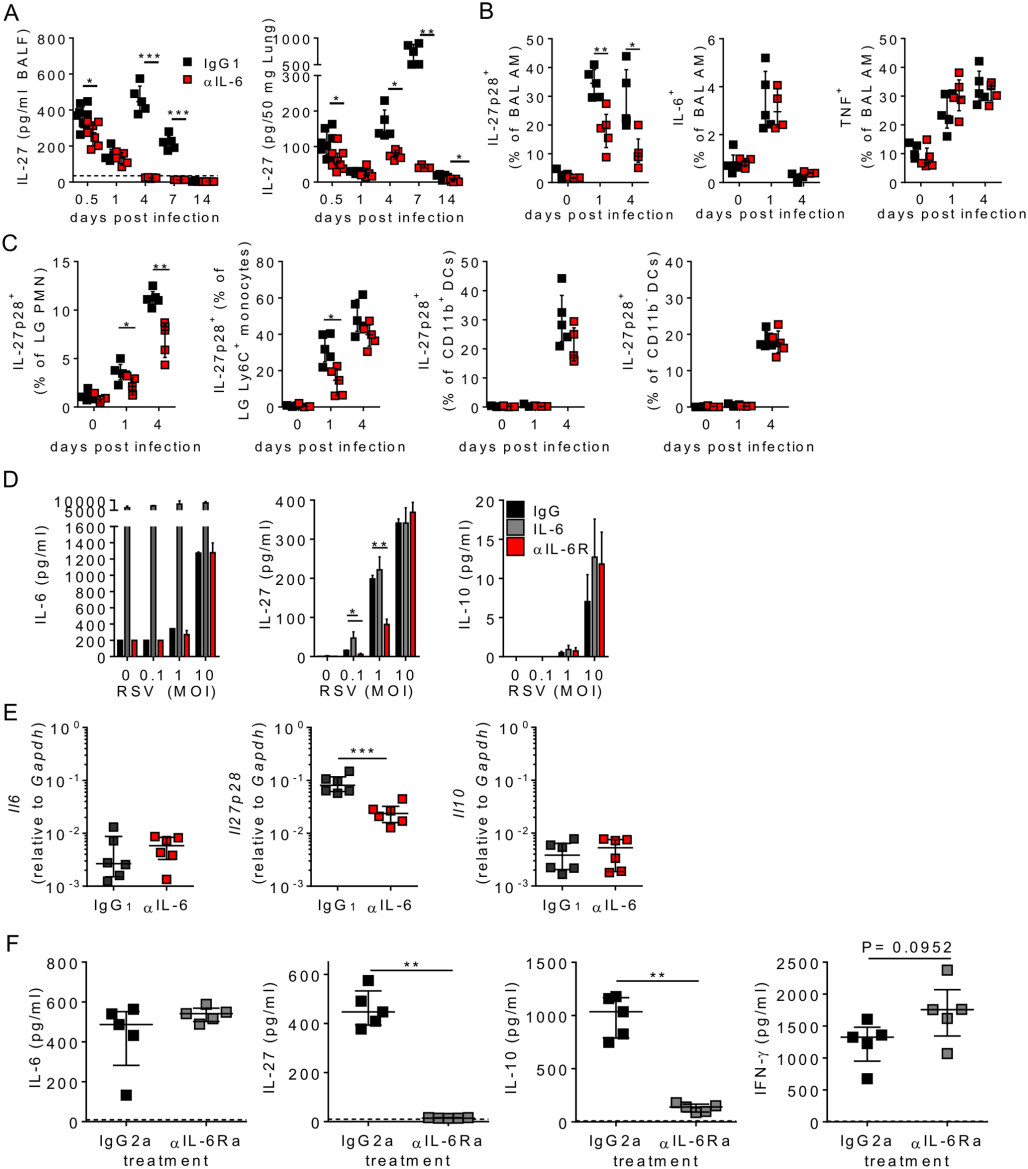
IL-6 upregulates IL-27 production in myeloid cells after viral exposure. (A-C) 8 week old BALB/c female mice were infected with 8 x 10^5^ ffu of RSV A2 and dosed with either αIL-6 or isotype control antibody i.p. between days -1 and 3 p.i. (A) IL-27 in the BAL and lungs after infection. (B) IL-27^+^, IL-6^+^ and TNF^+^ alveolar macrophages in the BAL. (C) IL-27^+^ neutrophils, Ly6C^+^ monocytes, CD11b^+^ and CD11b^-^ DCs in the lungs. Data is n = 5 mice per group and representative of 2 independent experiments. (D) IL-6 and IL-27 concentrations in the supernatant from primary murine alveolar macrophages at 24 hours p.i. with RSV A2 in the presence of 50 ng/ml rIL-6 or blocking αIL-6R. Data represent n = 3 repeats per condition and are representative of 3 independent experiments. (E) At day 4 p.i. BAL alveolar macrophages (Siglec F^+^ CD11c^+^ AF^+^) were FACS isolated from RSV infected mice and expression of *Il6*, *Il27p28* and *Il10* relative to *Gapdh* determined by RT-qPCR. (F) 8 week old BALB/c female mice were infected with 8 x 10^5^ ffu of RSV A2 i.n. and given either αIL-6R or isotype control antibody on day -1 p.i. At day 4 p.i. IL-6, IL-27, IL-10 and IFN-γ were measured in the airways by ELISA. Dotted lines on the graphs represent the concentrations observed in uninfected mice. Data except E is n = 5 mice per group, and representative of 2 independent repeats. F is from n = 6 mice combined from 2 independent repeats.

Importantly IL-27 was readily detected in the airways of RSV infected, but not uninfected, mice as early as 12 hours post infection, with peak of IL-27 seen in the airways and lungs of infected mice between day 4 and 7 p.i. (Fig 5A). Early depletion of IL-6 significantly reduced the concentration of IL-27 in the airways and lungs throughout infection (Fig 5A). IL-27 was also reduced in the airways of IAV infected mice after IL-6 depletion (S4B Fig). Cells of the myeloid lineage (e.g. macrophages, DCs and neutrophils) are known to be potent producers of IL-27 in response to pathogenic stimuli [44]. Fitting with this we found that alveolar macrophages, (gated as in S5A Fig), the first professional immune cell to encounter respiratory infections, rapidly upregulated IL-27 after infection of mice with RSV (Fig 5C and S5B Fig). Critically the proportion of IL-27^+^ alveolar macrophages in the bronchoalveolar lavage was significantly reduced at days 1 and 4 p.i. when IL-6 was depleted, but TNF and IL-6 production by the same cells was unaffected (Fig 5B and S5B Fig). Similar observations were made when alveolar macrophages from the lung tissue were analysed (S6A Fig). RSV infection is also associated with the recruitment of neutrophils, DCs and monocytes into the airways and lungs. In addition to alveolar macrophages, we found that neutrophils, Ly6C^+^ infiltrating monocytes and CD11b^+^ DCs all produced IL-27 in response to RSV infection (Fig 5C and S5C Fig). IL-27 production by both neutrophils and Ly6C^+^ monocytes, but not CD11b^+^ DCs nor CD11b^-^ DCs, was reduced in mice treated with anti-IL6 (Fig 5C). The numbers of alveolar macrophages, neutrophils, monocytes and DCs in the lungs did not change with IL-6 depletion (S6B Fig). Other lung populations including T, B and NK cells did not appear to produce significant IL-27 *in vivo*. As has previously been reported RSV infection results in alveolar macrophages upregulating MHCII (indicative of an M1-like phenotype). This was not affected by IL-6 depletion (S6C Fig). IL-27 production was limited to those alveolar macrophages that had also upregulated MHCII (S6D Fig).

*Ex vivo* murine alveolar macrophages produced IL-6, IL-27 and IL-10 in response to increasing doses of RSV (Fig 5D). Addition of recombinant IL-6 alone was not sufficient to induce IL-27 production by alveolar macrophages, but did enhance IL-27 secretion in combination with RSV. Accordingly, blocking IL-6R signalling during RSV exposure reduced IL-27, but not IL-6, secretion (Fig 5D). In contrast IL-10 production by alveolar macrophages after RSV exposure was not affected by either IL-6 addition or IL-6R blockade (Fig 5D). Accordingly, alveolar macrophages isolated from αIL-6 treated mice at day 4 p.i. showed significantly reduced expression of *Il27p28* compared to isotype treated mice (Fig 5E). *Il6* and *Il10* expression were both significantly lower than *Il27p28* expression at this timepoint and were unaffected by IL-6 depletion (Fig 5E).

Blocking IL-6R *in vivo* also resulted in significant decreases in IL-10 and IL-27, but not IL-6 nor IFN-γ, in the airways on day 4 post RSV infection (Fig 5F). Together this data showed that IL-6R signalling can directly promote the production of IL-27 by multiple myeloid cells types following RSV infection.

### IL-27 signalling in the airways regulates RSV specific T cell responses

**Fig 6:**
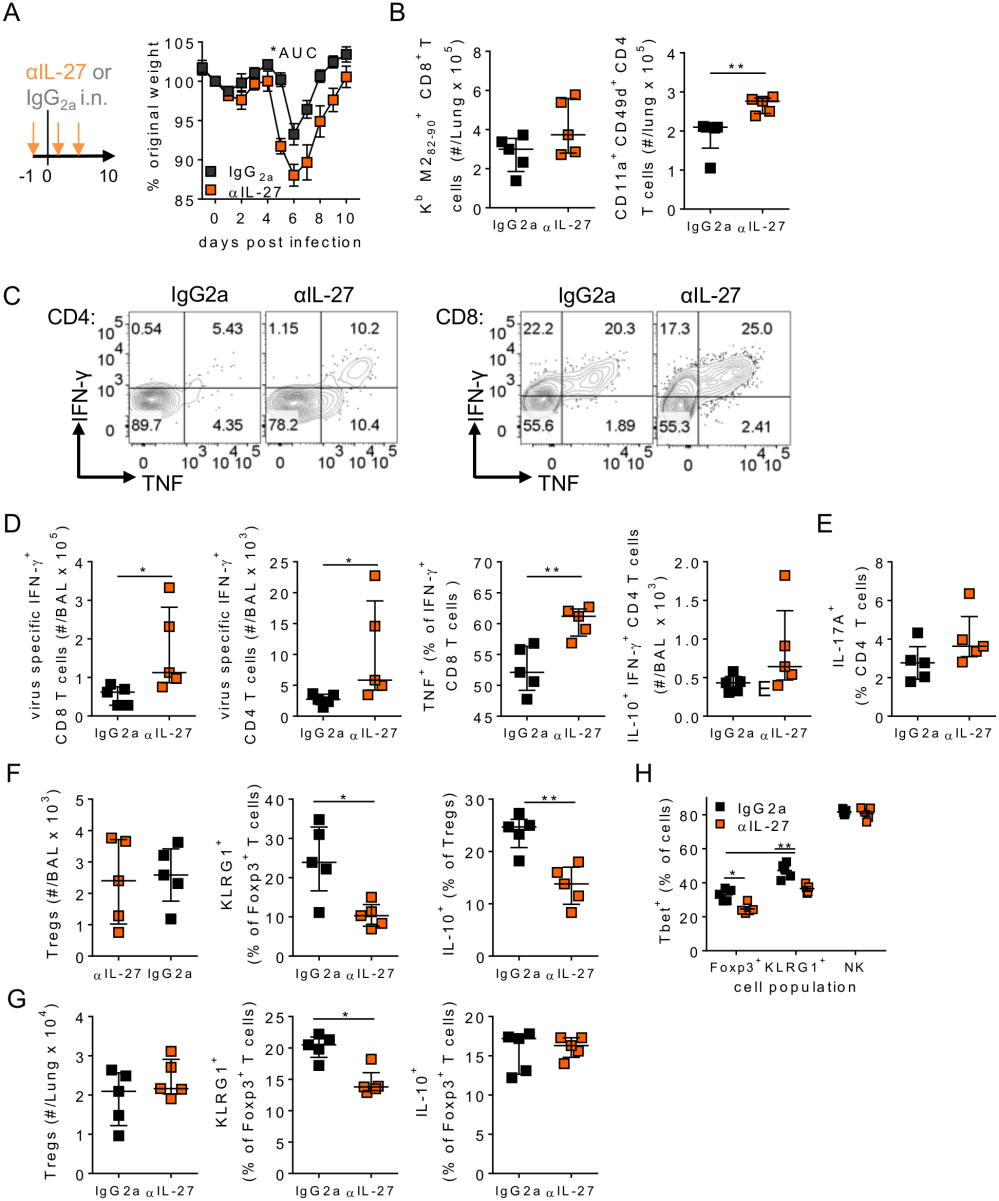
Airway IL-27 promotes virus specific T cell suppression in respiratory viral infection. 8 week old BALB/c mice were infected with 8 x 10^5^ ffu of RSV A2 and dosed with either αIL-27 or isotype control antibody i.n. between days -1 and 3 p.i. (A) Weight was measured daily. (B-H) At day 10 p.i. mice were euthanized and (B) Lung virus specific CD8 and CD4 T cell responses, (C-D) airway virus specific production of IFN-γ, TNF and IL-10 by CD4 and/or CD8 T cells, (E) IL-17A^+^ CD4^+^ T cells following polyclonal stimulation, (F) airway and (G) lung Treg numbers and expression of KLRG1^+^ and IL-10 and (H) T-bet expression by lung Tregs, KLRG1^+^ Tregs and natural killer (NK) cells were all measured. For Treg IL-10 measurements cells were stimulated with PMA/I in the presence of BFA prior to intracellular staining. Data represents n = 5 mice per group and is representative of n = 2 independent experiments.

To determine the role of IL-27 during RSV infection, we administered IL-27 neutralising antibodies into the airways prior to viral infection (Fig 6A). Lack of IL-27 early during RSV infection resulted in enhanced weight loss compared to isotype control treated mice, similar to that seen in the absence of IL-6 (Fig 6A). IL-27 depletion also enhanced virus specific CD8^+^ and CD4^+^ T cell numbers in the lungs and increased IFN-γ and TNF production by virus specific T cells in the airways, but there were similar numbers of IL-10^+^ Tr1 cells (Fig 6B–6D). As before IL-17A production by CD4^+^ T cells was not seen after virus specific stimulation, and after polyclonal stimulation was not different between isotype and αIL-27 treated animals (Fig 6E). Loss of IL-27 also reduced the numbers of activated Tregs (KLRG1^+^) in the airways and Treg derived IL-10 (Fig 6F). Similar data was seen in the lungs (Fig 6G). IL-27 has been shown to upregulate the canonical T_H_1 transcription factor T-bet in a number of different lymphocyte populations including Tregs [42, 44]; Lung KLRG1^+^ Tregs had enhanced expression of T-bet compared to normal Tregs, and expression of T-bet in both was downregulated in the absence of IL-27, however T-bet expression in lung NK cells was unaffected by the anti-IL-27 treatment (Fig 6H). Together this showed that production of IL-27 in the airways after respiratory virus infection plays a similar role to that of IL-6 and is critical for promoting local Treg function, dampening virus specific T cell responses and weight loss.

### IL-27 is sufficient to promote IL-6 dependent immunoregulation

**Fig 7:**
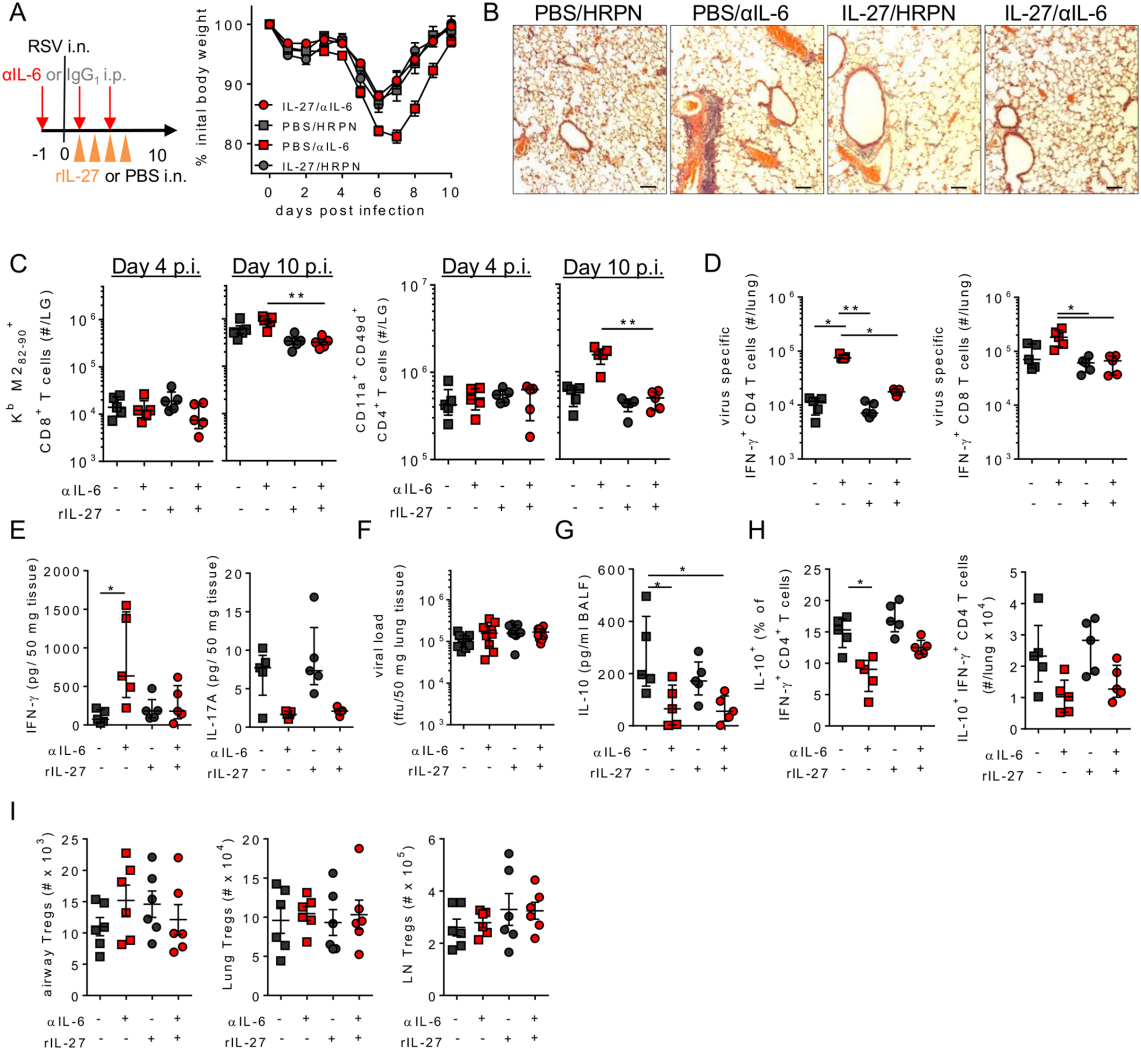
IL-27 promotes IL-6 dependent resolution of RSV disease. 8 week old BALB/c female mice were infected with 8 x 10^5^ ffu of RSV A2 and dosed with either αIL-6 or isotype control antibody i.p. between days -1 and 3 p.i. (A) Mice received either rIL-27 or PBS i.n. on days 1–4 p.i. and were weighed until day 10 p.i. (B) Representative H&E staining of lung tissue using a 10X objective at day 10 p.i., black bar represents 200 μm. (C) virus specific CD4^+^ and CD8^+^ T cells in the lung were enumerated at days 4 and 10 p.i. (D) At day 10 p.i. IFN-γ^+^ CD4 and CD8 T cells in the lungs following RSV peptide simulation and (E) IFN-γ and IL-17A in lung homogenate were determined. At day 4 p.i. (F) Lung viral load, (G) IL-10 in the airways, (H) IL-10^+^ Tr1 cells and (I) Foxp3^+^ Tregs in the BAL, lungs and lung draining lymph nodes were determined. (A-E) Data representative of n = 5 mice per group from 2 independent experiments. (F) Represents n = 10 mice per group combined from 2 independent repeats. (G-I) Data represents n = 6 mice pooled from 2 independent repeats.

We next assessed if IL-6 regulated RSV mediated pathology via its induction of IL-27. Administration of IL-27 into the airways of virally infected mice completely abrogated the enhanced disease seen on depletion of IL-6, while administration of IL-27 alone had no effect on RSV mediated weight loss (Fig 7A). Fitting with the changes in weight loss αIL-6 depletion resulted in more cellular aggregation around the blood vessels and airways in the lungs of RSV infected mice at day 10 p.i. compared to isotype treated controls. This was reduced in mice that received IL-27 (Fig 7B). In addition, recovery from enhanced disease was associated with reduced virus specific CD4^+^ and CD8^+^ T cells at day 10 p.i. in IL-27 treated mice compared to those which received αIL-6 alone, with frequencies similar to mice with intact IL-6 signalling (Fig 7C). Similar findings were observed when analysing virus specific IFN-γ^+^ CD4^+^ and CD8^+^ T cells at day 10 p.i. (Fig 7D). IL-6 depletion increased the concentration of IFN-γ in the lungs, and decreased IL-17A concentrations, while IL-27 treatment dampened IFN-γ concentrations, fitting with the observed changes in virus specific T cell responses, but did not restore IL-17A (Fig 7E).

IL-27 has been reported to have direct anti-viral properties, which could limit T cell responses [45, 46], however local administration of IL-27 did not affect peak RSV titres in the lung, irrespective of the presence of IL-6 (Fig 7F). To determine if IL-27 was suppressing RSV mediated immunopathology through any of the regulatory T cell subsets we next analysed their activation or recruitment at day 4 p.i., immediately after the last dose of IL-27 was administered, but prior to the onset of weight loss. Neither airway IL-10 nor polyclonal Tr1 cells were significantly upregulated by IL-27 treatment compared to mice that received αIL-6 alone (Fig 7G & 7H), nor were the number of Tregs in the airways, lungs or lymph nodes changed by rIL-27 treatment (Fig 7I).

### IL-27 promotes molecules associated with Treg suppressor function

**Fig 8:**
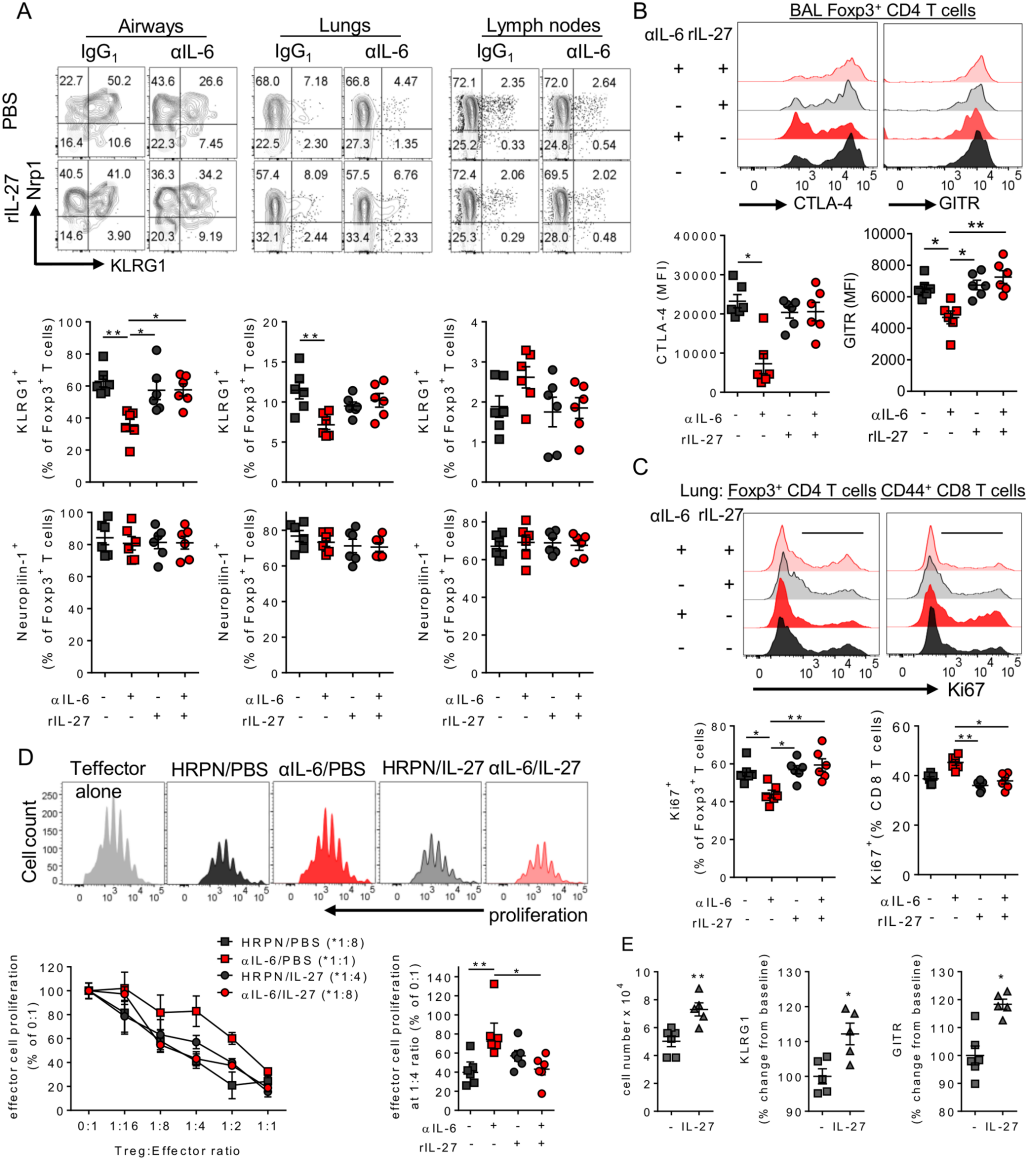
An IL-6/IL-27 dependent pathway matures regulatory T cells after RSV infection. 8 week old BALB/c female mice were infected with 8 x 10^5^ ffu of RSV A2 and dosed with either αIL-6 or isotype control antibody i.p. between days -1 and 3 p.i. (A) Treg expression of KLRG1 and Neuropilin alongside (B) CTLA-4 and GITR in the airways and (C) Ki67 expression in the lungs. (D) CD4^+^ GITR^+^ CD25^+^ Tregs were FACS isolated from BAL and lungs at day 4 p.i. and co-cultured in increasing concentrations with proliferation dye stained, activated naïve splenic CD4^+^ T cells for 5 days. Proliferation of these “effector” T cells, relative to effector CD4 T cells cultured without Tregs, was then calculated. Representative plots from the 1:4 Treg:Effector cell ratio are depicted. The dilution at which Tregs from each condition significantly suppressed effector cells compared to effector cells on their own is shown in brackets in the legend. (E) CD25^+^ splenic Tregs were activated *in vitro* with αCD3/28 in the presence or absence of 50 ng/ml of rIL-27 and KLRG1 and GITR expression were measured 48 hours later. (A-D) Data represents n = 6 mice pooled from **2** independent repeats. (E) represents 5 mice from 3 independent experiments.

While IL-27 treatment failed to increase Treg frequencies prior to disease onset it did appear to restore Treg maturation in the respiratory tract of mice deficient in IL-6 to proportions seen in control treated mice (Fig 8A). KLRG1^+^ Tregs were particularly frequent in the airways, with the fewest seen in lymphoid tissue, and the majority were neuropilin^+^ indicating thymic origin (Fig 8A and S7A Fig). Further characterization of the airway Tregs after RSV infection showed they had high expression of CTLA-4, GITR, Lag-3 and IL-10 amongst other important regulatory molecules in comparison to the Tregs found in the lungs and LNs (S7B Fig). Alongside reduced KLRG1 expression, depletion of IL-6 also caused reduced expression of both CTLA-4 and GITR by airway Tregs after RSV infection, which was restored by IL-27 treatment (Fig 8B). In addition while nearly all airway Tregs were proliferating, as measured by Ki67, and this did not change with treatment (S6B Fig), proliferation of lung Tregs was significantly reduced by αIL-6 treatment and restored by rIL-27 treatment (Fig 8C). Inversely, there was increased proliferation of CD8^+^ T cells in the lungs in the absence of IL-6 which was suppressed by IL-27 administration (Fig 8C). By day 10 p.i., when all mice had recovered from weight loss but still had heightened CD8^+^ T cell responses, there was still no observable difference in Treg number the lungs (S7C Fig). There was however still a trend for Tregs from IL-6 depleted mice to have lower frequencies of KLRG1 (S7C Fig).

Importantly CD25^+^ GITR^+^ CD4 T cells (which were found to be at least 95% Foxp3^+^) isolated from the lungs and airways of IL-6 depleted mice at day 4 p.i. were also less suppressive than CD25^+^ GITR^+^ CD4 T cells isolated from isotype treated mice in an *in vitro* suppression assay (Fig 8D). *In vivo* IL-27 treatment restored Treg suppressive capacity, in the absence of IL-6. *In vitro* culturing CD4^+^ CD25^+^ splenic Tregs from uninfected mice in the presence of rIL-27 was sufficient to increase Treg numbers and expression of GITR and KLRG1 compared to cultures without rIL-27 (Fig 8E). Of note, Lag3 was not upregulated in these cultures, despite this having been reported previously [43]. This data showed loss of IL-6 during RSV infection impaired the expression of functional molecules on Tregs in the respiratory tract and their suppressive capacity. This could be restored by exogenous treatment with IL-27.

### IL-27 can promote disease resolution in an IL-10R independent manner

**Fig 9:**
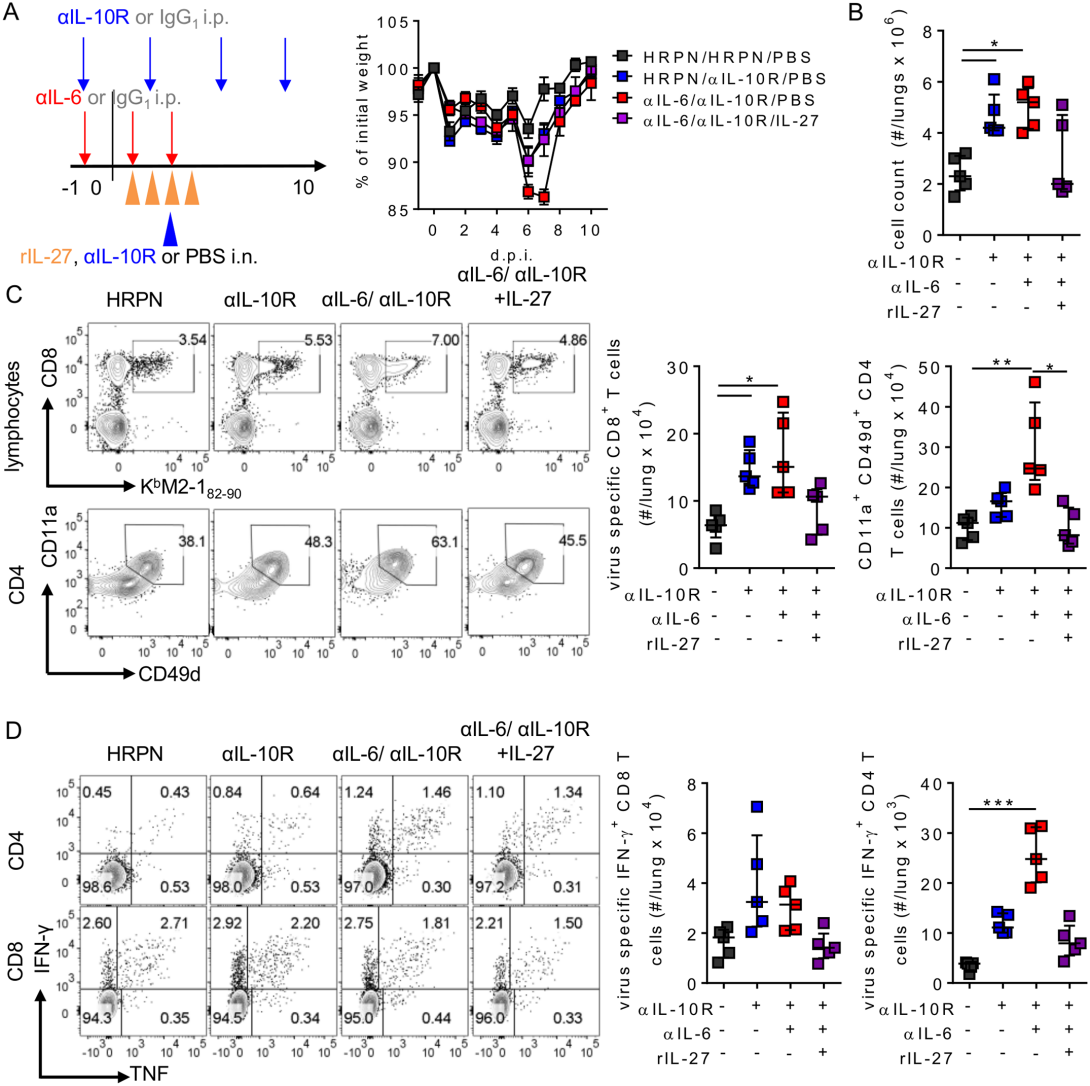
IL-27 acts independently of IL-10R signalling to regulate weight loss and T cell responses to RSV. 8 week old BALB/c mice were dosed as in Fig 7, and in addition were dosed with αIL-10R i.p. on days -1, 2, 5 and 8 p.i. and i.n. on day 3 p.i. or isotype control. Mice were then infected with 2 x 10^5^ ffu of RSV A2 on day 0. (A) Schematic of dosing and weight change over time following infection. At day 10 p.i. the (B) total lung cell counts, (C) representative flow plots and enumeration of RSV tetramer specific CD8^+^ T cells and antigen experienced CD4^+^ T cells in the lungs and (D) IFN-γ^+^ and TNF^+^ CD8 and CD4 T cells in the lungs following RSV peptide stimulation were determined. Data represents n = 5 mice in each group and representative of 2 independent experiments.

As IL-27 treatment rescued IL-6 deficient mice from enhanced pathology without fully restoring IL-10 concentrations, we wished to establish whether IL-6 dependent immune regulation required IL-10 signalling. We therefore treated mice with blocking αIL-10R, as has previously been reported [22], in the presence or absence of αIL-6. Mice were then infected with a low dose of RSV A2, sufficient to induce approximately 5% weight loss at day 6 p.i. in isotype treated control mice in order to avoid excess morbidity and mortality. Fitting with previous literature, IL-10R blockade resulted in increased weight loss and delayed resolution compared to isotype treated mice (Fig 9A). Importantly αIL-6 treatment further increased this weight loss, while administration of IL-27 resulted in αIL-6/αIL-10R having similar weight loss to that seen in αIL-10R treatment alone (Fig 9A). Fitting with this, lung cell counts at day 10 p.i. were significantly increased in mice receiving either αIL-10R with or without αIL-6 in comparison to isotype treated control mice, while IL-27 treatment reduced this (Fig 9B). In addition virus specific CD8^+^ and CD4^+^ T cells in the lungs were both elevated in αIL-10R treated mice, and virus specific CD4^+^ T cells were further enhanced by combined IL-6 depletion (Fig 9C). IL-27 administration to αIL-10R and αIL-6 treated mice resulted in virus specific T cell numbers in the lungs comparable to those seen in the control mice (Fig 9C). Similar results were seen after peptide stimulation with increased IFN-γ^+^ CD4^+^ and CD8^+^ T cells present after treatment with αIL-10R and αIL-6, which could be corrected by co-treatment with IL-27 (Fig 9D). Taken together this showed that IL-27 could act even in the absence of IL-10R signalling.

## Discussion

Respiratory viruses such as RSV elicit a rapid host inflammatory response from the respiratory stroma and resident immune cells such as alveolar macrophages. One of the hallmark mediators associated with this response is the pro-inflammatory cytokine IL-6. Here we find that early production of IL-6 is essential in promoting the regulation of immune responses after viral infection of the respiratory tract. IL-6 is critical in enhancing the production of IL-27 by both resident and infiltrating myeloid cells after infection. Local IL-27 then promotes the maturation of regulatory T cells in the lungs and curbs virus specific T_H_1 mediated immunopathology, without affecting viral clearance.

High concentrations of IL-6 are regularly seen after severe respiratory infection, and are often considered an indicator of un-checked disease [47]. In contrast, low production polymorphisms in the *Il6* gene are actually associated with increased risk of disease in both rhinovirus and RSV infection [27]. Clinical targeting of the IL-6 pathway is used to treat a number of inflammatory disorders, in particular rheumatoid arthritis [1]. One of the most common side effects of targeting either the IL-6R (via Tocilizumab) or IL-6 (via Siltuximab) is increased risk of both upper and lower respiratory tract infections [48, 49]. We find that IL-6 is produced rapidly upon respiratory infection, but that elevated concentrations are seen throughout infection. Importantly we found that IL-6 produced early after infection limited disease severity, but that IL-6 signalling occurring at the peak of disease had little, or limited effect on the outcome of infection. This highlights the critical role of timing in determining the role of mediators such as IL-6 in the outcome of disease. Indeed, while RSV induces IL-6 rapidly *in vitro* and *in vivo* in both in mouse models and humans, experimental human challenge infections find that it takes up to a week to see peak symptoms [50]. Heightened IL-6 measurements seen in patients hospitalized with severe respiratory infection are likely to reflect increased inflammation, but not indicate elevated IL-6 production at the earliest stages of infection, where our data indicates it might play a protective role.

IL-6 has a multitude of pro-inflammatory roles in the immune system, promoting the survival and recruitment of neutrophils, the proliferation of both T and B cells, and the differentiation of T_H_17 cells. It is therefore noteworthy that in the context of both RSV and IAV infection we found that IL-6 suppresses effector T cell responses and overall inflammation in the airways and lungs. While the role of IL-6 in RSV infection has not previously been studied, use of IL-6 deficient mice has shown that IL-6 is critical in controlling viral load after infection with either an H5N1 or H1N1 influenza A virus, with mice succumbing to lethal infection in IL-6’s absence [12, 14]. In the case of H5N1 IAV IL-6 promotes the survival of anti-viral neutrophils at the site of infection [12]. Depletion of neutrophils during RSV infection does lead to slightly increased peak viral titres although viral clearance is unaffected [51]. Given we saw no difference in neutrophilia or peak viral loads this suggests that IL-6 is redundant for neutrophil maintenance during RSV infection.

More markedly, infection of IL-6 deficient mice with a similar, sub-lethal dose of H1N1 PR8 to the one used in this study results in a reduced proliferation of anti-viral T cells, and their failure to accumulate in sufficient numbers [14]. Here with both RSV and PR8, we found the opposite result, with increased virus specific T cell proliferation. A number of possibilities exist for the disparate outcomes in the absence of IL-6. For instance we utilized the BALB/c mouse strain for our studies, which show greater susceptibility to respiratory viral infections, especially RSV (reviewed in [52]), whereas IL-6 deficient mice are on the more resistant C57BL/6J background. In addition, germline deletion results in the absence of IL-6 throughout development while αIL-6 or αIL-6R antibodies remove signalling only at the point of infection. IL-6 is known to be involved in number of homeostatic processes including the regulation of hematopoietic stem cells and metabolism [53, 54], thus the long term absence of IL-6 may result in distinct immunological outcomes, compared to acute ablation.

IL-6 signalling is mediated by its binding to the cytokine specific receptor IL-6R and subsequent heterodimerization with the common IL-6 family signal transduction molecule, gp130. It can, however signal via either a classical pathway, whereby IL-6R on the surface of the target cell binds IL-6, or a trans-signalling pathway, where soluble IL-6R binds IL-6, then becomes membrane bound alongside gp130 to cause signalling [1]. Both classical and trans-signalling are blocked by IL-6 neutralizing or IL-6R blocking antibodies *in vivo*. Recently however IL-6 signalling has also been shown to occur via ‘trans-presentation’, with IL-6/IL-6R complexes being provided directly from one cell, to another, target cell [55]. This form of IL-6 signalling is not thought to be responsive to αIL-6 antibodies, though αIL-6R blockade may be more effective [55]. Trans-presentation was shown to be important in promoting T_H_17 responses in auto-immune disease, and could therefore be an important contributor to T cell responses in other inflammatory settings. If the use of αIL-6 or αIL-6R antibodies in this study does not block trans-presentation this may also help to explain the differences between our findings and those made in *Il6* deficient mice.

Previous studies have found that IL-6 limits disease severity after infection by limiting pathogen burden [8, 12–14, 56]. Importantly here we found that IL-6 mediated immune-regulation occurs via the induction of IL-27 in the airways after infection, without any effect on peak viral loads or viral clearance. IL-27 compensates for the loss of IL-6, and IL-27 depletion recapitulates the phenotype seen in the absence of IL-6. IL-27 is a potent immune-regulatory cytokine capable of dampening inflammation in a broad range of diseases including malaria, *Toxoplasma gondii* and IAV infections, inflammatory arthritis and experimental allergic encephalomyelitis [57–61]. IL-27 acts through a number of mechanisms to limit inflammation, including negatively regulating T_H_17 differentiation [57], promoting inhibitory ligands such as CD39 and PD-L1 [59, 62], promoting IL-10 production by T cells [63] and promoting regulatory T cell activity [42]. IL-27 dampens inflammation and mucus inducing T_H_17 responses seen after infection with RSV line 19 [64]; However, while RSV line 19 is known to induce potent IL-17 responses *in vivo*, RSV A2 does not, and depleting or treating with IL-27 locally does not appear to affect the frequency of IL-17A^+^ CD4 T cells. We also detected no difference in DC expression of CD39 or PD-L1 in any of the conditions studied here. Instead, as found in *T*. *gondii* infection, collagen induced arthritis and cell-induced colitis [42, 43, 65, 66], IL-27 promotes the activation and proliferation of Tregs, especially in the airways, in murine RSV infection. In particular it enhances the expression of key suppressive molecules CTLA-4, GITR and KLRG1 (reviewed in [67]). We found IL-27 also up-regulates T-bet expression in Tregs during RSV infection, which is a critical controller of Treg function and homeostasis in T_H_1 associated immune responses [68], and this likely contributes to IL-27 mediated Treg maturation during RSV infection.

IL-6 depletion also causes significant ablation of IL-10 in the lungs after RSV infection, dampening Tr1 derived IL-10 production without seeming to affect macrophage derived IL-10. IL-10 is critical in determining the outcome of RSV infection [21, 22, 69], with CD4^+^ T cells thought to be the major source [21], and IL-27 has been shown to regulate IL-10 production by Tr1 cells and Tregs after IAV, murine cytomegalovirus and *T*. *gondii* infection [60, 63, 69, 70]. IL-27 does also partially regulate IL-10 production during RSV infection, but did not completely restore IL-10 production after IL-6 depletion. This indicates that IL-6 may promote several non-redundant pathways to induce IL-10. Indeed IL-6 can induce CD4^+^ T cell production of IL-21 [10, 71], and IL-21 is known to promote IL-10 [72]. Importantly however IL-27 was sufficient to dampen the immunopathology seen in the absence IL-6 even in the absence of IL-10R signalling. This suggests IL-27’s ability to promote enhanced immunosuppressive activity across a number of regulatory T cell subsets in the lungs and airways is more important than its specific ability to induce IL-10.

Overall, our data highlights an important and hitherto underappreciated role for IL-6 as a mediator of immune resolution after respiratory viral infection. Interestingly IL-6 mediates resolution by inducing the production of the immunoregulatory cytokine IL-27 by myeloid cells in the lungs and airways of infected individuals. IL-27 mediates suppression of pathogenic immune responses and enhances the local maturation of regulatory T cell populations. Further evaluation of the IL-6/IL-27 axis may shed light on the factors critical in determining immune resolution, and help us develop novel therapeutic strategies for severe disease.

## Materials and methods

### Ethics statement

Mice were housed in IVCs and all procedures were approved by the Imperial College London Animal Welfare Ethical Review Body (AWERB) and the United Kingdom Home Office (Approval from both under project licence number 70/7463) and conducted in accordance with the Animals (Scientific Procedures) Act 1986. Mice were anesthetized via inhalation of isoflurane and euthanized via intraperitoneal overdose of pentobarbitone, using exsanguination via a peripheral vein as a secondary means of confirmation.

### Animals and viral infections

Specific pathogen free 6–7 week old female BALB/c mice were purchased from Charles River (Margate, UK). Plaque purified RSV A2 (obtained from ATCC) was grown in HEp-2 cells, and viral titre determined by focus forming assay as described [25]. H1N1 IAV PR8 (strain A/Puerto Rico/8/1934 H1N1) was kindly provided by Professor Wendy Barclay (Imperial College London, UK). At 8 weeks of age mice were intranasally (i.n.) infected with 8 x 10^5^ ffu of RSV in 100 μl or 40 plaque forming units (pfu) of IAV PR8 in 50 μl whilst under isofluorane anesthesia. Weight and symptoms was measured daily to monitor disease severity. Symptom scoring was carried out by scoring mice from 0–2 for each of the following: piloerection, hunched posture and inactivity. Mice were deemed to have reached their humane endpoint if weight loss exceeded 25% of bodyweight on 2 consecutive days in accordance with our Home Office licence.

### *In vivo* treatments

Rat anti-mouse IL-6 (MP5-20F3), anti-mouse IL-6R (15A7), anti-mouse IL-10R (1B1.3A) and their isotype controls IgG_1_ (HPRN) and IgG_2b_ (LTF-2) were purchased from BioXCell (West Lebanon, NH). Rat anti-mouse IL-27p28 (MM27-7B1), rat IgG_2a_ (MOPC-173) and recombinant mouse IL-27 (rIL-27) were purchased from Biolegend (San Diego, CA). MP5-20F3 and HPRN were administered i.p. in 200 μl PBS (0.5 mg on day -1 p.i., and 0.25 mg every 2 days from day 1 p.i.). 15A7 and LTF-2 were administered i.p. in 200 μl PBS (150 μg on day -1 p.i.). MM27-7B1 and MOPC-173 were administered i.n. in 100 μl PBS (50 μg every other day from day -1 p.i.). rIL-27 was delivered i.n. in 100 μl (200 ng daily from days 1–4 p.i.). 1B1.3A was administered in 200 μl PBS i.p. (0.25 mg on days -1, 2, 5 and 8 p.i. and in 100 μl PBS i.n. (0.15 mg on day 3 p.i.).

### Histology

For immunohistochemistry, lungs were inflated with 1 ml of 10% formalin via the trachea, and the lungs were then tied off. After overnight fixation in 10% formalin the lung tissue was then paraffin embedded and 4 μm sections stained with hematoxylin and eosin (H&E) stained. Sections were then imaged by light microscopy.

### Cell recovery

For bronchoalveolar lavage (BAL) for cells and supernatants the trachea was cannulated and the lungs washed with 1 ml PBS a total of 3 times; after centrifugation supernatant was stored for measurement of mediators and cells used for flow cytometry and H&E staining. The left lung lobe was snap frozen in liquid N_2_, and subsequently homogenized on ice with a rotor-stator; whole homogenate was used for viral load measurements, and the supernatant taken for mediator analysis. The remaining lung tissue and mediastinal lymph nodes (LN) were taken for flow cytometry. Lung tissue was weighed, diced and incubated for 45 mins at 37°C in complete RPMI containing RPMI 1640, 10% FCS, 2 mM L-glutamine, 100 U/ml penicillin/streptomycin (Life Technologies), 0.15 mg/ml collagenase D (Roche Diagnostics) and 25 μg/ml Dnase I (Roche Diagnostics). Lung and LN tissue was then passed through 100-μm-mesh-size cell strainers (BD Pharmingen) and washed through with a 5 ml volume of RPMI 1640. After the removal of the supernatants, cells were treated with ACK lysing buffer (150 mM ammonium chloride, 10 mM potassium bicarbonate, 0.1 mM EDTA) Finally, they were resuspended in complete RPMI. Cell viability was assessed by trypan blue exclusion, and total cell numbers were counted by use of a disposable multiwell hemocytometer (Immune Systems, United Kingdom).

### Flow cytometry

Flow cytometry was carried out as described in [71]. 1–2 x 10^6^ and 3–4 x 10^6^ single cells were taken for extracellular or intracellular stains respectively. Cells were stained with fixable live/dead dyes (Biolegend) and prior to surface staining were incubated in 1 in 100 dilution of anti-mouse TruStain-FcX (Biolegend) for 10 minutes. K^b^ M2-1_82−90_ containing monomers were kindly provided by the NIH Tetramer Core facility, and folded with SA-PE (Molecular Probes, Invitrogen) in the lab. The following anti-mouse antibodies (from Biolegend unless otherwise stated) were then used for surface staining: anti-CD4 (RM4-5), -CD3e (145-2C11, eBioscience, Life Technologies), -CD8 (53–6.7), -B220 (RA3-6B2), -CD19 (6D5), -CD11c (N418, eBioscience), -CD11b (M1/70), -Ly6C (HK1.4), -Ly6G (1A8), -CD45 (30-F11), -CD90.2 (53–2.1), -Nkp46 (29A1.4), -CD64 (X54-5/7.1), -Ia/Ie (M5/114.15.2), -CD11a (M17/4), -CD49d (R1-2, eBioscience), -CD44 (IM7), -CD62L (MEL-14), -PD-1 (29F.1A12), -KLRG1 (MAFA), -LAG3 (C9B7W, eBioscience), -GITR (DTA-1, BD Biosciences), -CD25 (PC61) and -Neuropilin-1 (3E12). For intracellular cytokine staining cells were fixed with 1% paraformaldehyde (Sigma Aldrich) in PBS for 10 minutes at room temperature and then the following antibodies stained for using permeabilization buffer (eBioscience): anti mouse-IFN-γ (XMG1.2), -TNF (MP6-XT22), -IL-17A (TC11), -IL-13 (eBio13A, eBioscience), -IL-4 (11B11), -IL-10 (JES5-16E3), -IL-6 (MP5-20F3), -IL-27 (MM27-7B1), and anti-human Granzyme B (GB11, eBioscience). For intranuclear staining cells were fixed using Foxp3 staining buffer (eBioscience) following manufacturers’ instructions and the following antibodies were used: anti mouse-Foxp3 (FJK-16s, eBioscience), -RORγt (AFKJS-9, eBioscience), -Helios (22F6), -T-bet (4B10), -CTLA4 (UC10-4B9) and -Ki67 (16A8). Samples were acquired on a 5-laser, 18-parameter BD Fortessa and analyzed using FlowJo (TreeStar, Ashland, OR).

### *Ex vivo* stimulations for intracellular cytokine staining

For T cell cytokine staining cells were first stimulated for 5 hours with 10 ng/ml PMA and 0.5 μg/ml of ionomycin (Sigma-Aldrich), 2 μg/ml of CD8 stimulating RSV M2-1_82−90_ (SYIGSINNI) peptide or 5 μg/ml of pooled CD4 stimulating RSV G_181-197_ (TCWAICKRIPNKKPGKK), F_51-66_ (GWYTSVITIELSNIKE) and P_39-55_ (SIISVNSIDIEVTKESP) peptides in the presence of 10 μg/ml Brefeldin A (Sigma Aldrich) and 50 U/ml recombinant mouse IL-2 (R&D Systems, Minneapolis, NE). For IL-27, TNF and IL-6 production cells were incubated in the presence of BFA alone for 5 hours. Unstimulated and no BFA control samples showed no positive cytokine staining and were used for the purposes of gating.

### *In vitro* Treg suppression assay

CD4^+^ T cells were enriched from lung and BAL cells using a negative selection CD4 enrichment kit per manufacturers’ instructions (StemCell). They were then stained with antibodies against CD4, GITR and CD25 alongside Topro3 as a viability dye. Pools of 100,000 live CD4^+^ GITR^+^ CD25^+^ cells were then sorted to >95% purity on a BD Aria III under Bsl2 conditions. Naïve CD4^+^ T cells were isolated from the spleens of uninfected mice using a mouse naïve CD4^+^ T cell isolation kit according to manufacturers’ instructions (StemCell). They were then stained with Tag-it Violet (TV) proliferation and cell tracking dye (Biolegend). 10,000 labelled naïve CD4^+^ T cells were then co-cultured with increasing ratios of CD4^+^ GITR^+^ CD25^+^ up to 1:1 in the presence of 30 U/ml rIL-2 and Mouse activator CD3/28 Dynabeads (ThermoFisher) in 96-well U bottom tissue culture plates (Corning). The cells were then incubated for 5 days in the dark at 37°C. Cells were then washed with PBS and stained with far-red fixable viability dye (ThermoFisher) and resuspended in 200 μl of FACS buffer. Each well was then run at a constant speed and time on a flow cytometer. The number of viable, proliferating effector cells (viability dye negative, TV diluted) in each well was then calculated. Percent proliferating effector cells was calculated versus naïve CD4^+^ T cells stimulated in the absence of Tregs.

### *Ex* vivo macrophage and Treg cultures

Murine alveolar macrophages were isolated by cannulating the trachea and flushing the airways with 1 mL PBS containing 5 mM EDTA 10 times (total yield 10 mL). Macrophages were purified by adhering to culture plates for 2 hours, then stimulated with increasing titres of RSV at 0, 0.1, 1 or 10 mean of infectivity (MOI) in RPMI containing 100 U/ml penicillin/streptomycin and 2 mM L-glutamine (Life Technologies), either alone or with the addition of 50 ng/mL recombinant mouse IL-6 (Biolegend) or 10 μg/mL anti-mouse IL-6R (BioXCell). All culture supernatants were harvested 24 hours post infection. Aveolar macrophages (Siglec F^+^, CD11c^+^, Autofluorescent^+^) from infected mice were FACS isolated from the BAL using a BD FACS Aria III under Bsl2 conditions, and cell pellets immediately lysed in RLT. RNA was then isolated using RNeasy micro-columns (Qiagen) and converted to cDNA using the GoScript RT kit (Promega) according to manufacturers’ instructions. *Il10*, *Il27*, *Il6* and *Gapdh* (Primers and probe sets from Applied Biosystems) expression was then determined by taqman qPCR on a Viia7 qPCR machine (Applied Biosystems). Splenic CD25^+^ Tregs were isolated to 90–95% purity from uninfected mice using the EasySep mouse CD25 regulatory T cell selection kit (Stemcell Technologies, Cambridge, UK) according to manufacturers’ instructions and stimulated as previously described [43]. Briefly they were stained with CFSE to track cell proliferation (Biolegend) and stimulated with Mouse T-activator CD3/28 Dynabeads and 30 U/ml rIL-2 in the presence or absence of 50 ng/ml of rIL-6 or rIL-27 for 48 hours.

### Cytokine measurements

Mouse IL-6, IL-10, IFN-γ and IL-27 were determined using Ready-SET-Go ELISA kits (eBioscience) according to manufacturers’ instructions.

### Statistics

Graphpad 6.0 (Graphpad, San Diego, CA) was used for all statistics. For weight loss area under the curve (AUC) was calculated and significance tested by Mann-Whitney U test. For experiments with only 2 groups, non-parametric Mann-Whitney U test was used. For groups with 3 or more groups, non-parametric Kruskal-Wallis H test with a Dunn’s test were used. In all cases * P < 0.05, ** P < 0.01 and *** P < 0.001.

### Data availability

The data that support the findings of this study are available from the corresponding author on request.

## Supporting information

S1 Fig Anti-IL-6 treatment depletes RSV induced IL-6 both locally and systemically. 8 week old BALB/c mice were infected with 8 x 10^5^ ffu of RSV A2 i.n.. Mice were treated with anti-IL6 or IgG_1_ isotype control i.p. from days -1 to 13 p.i. IL-6 was measured by ELISA in the BAL, lung homogenate and serum at the indicated days post infection. Data is representative of n = 2 independent repeats of n = 5 mice per time point. Kruskal-Wallis H test was carried out between baseline and each d.p.i.
(PDF)

S2 Fig IL-6 regulates the proportion of virus specific CD8 T cells. 8 week old BALB/c mice were infected with 8 x 10^5^ ffu of RSV A2 i.n. and given 0.5 mg of either HRPN (IgG_1_) or MP5-20F3 (αIL-6) i.p. on day -1 p.i. and 0.25 mg i.p. every other day after that. Mice were euthanized at days 4, 7 and 14 p.i. Flow cytometry was used to determine the proportion of K^b^M2_82-90_^+^ CD8^+^ T cells in the lungs **(A)** and lymph node **(B)**. Data is representative of 5 mice per group and 2 independent repeats. Plots depict the median percentage tetramer positive cells within the total lymphocyte population for each group at each time point.
(PDF)

S3 Fig Early, but not late, IL-6 signalling regulates RSV induced disease. 8 week old BALB/c female mice were infected with 8 x 10^5^ ffu of RSV A2 i.n. and dosed with either αIL-6 or isotype control antibody as shown in Fig 5A. Clinical symptom scores were taken daily. Data are representative of n = 5 mice per group and 2 independent experiments. Area under the curve (AUC) was calculated and Mann-Whitney test between control and αIL-6 treated groups for each regime carried out.
(PDF)

S4 Fig IL-6 regulates disease resolution after influenza A virus infection. 8 week old BALB/c female mice were infected with 40 pfu of IAV PR8 and dosed with either αIL-6 or isotype control antibody i.p. between days -1 and 3 p.i. **(A)** Weight loss was monitored daily, area under the curve (AUC) was used to test statistical significance. **(B-H)** Mice were euthanized at day 10 p.i. and **(B)** IL-6, IL-10 and IL-27 in the BAL and **(C)** IFN-γ in the lungs were measured by ELISA. **(D)** The frequency of antigen experienced CD8^+^ T cells (PD1^+^CD44^+^CD62L^-^) and CD4^+^ T cells in the lungs. **(E)** The frequency of lung IFN-γ^+^ CD4 T cells in the lungs, and **(F)** the proportion that were IL-10^+^ after PMA/I stimulation. **(G)** Foxp3^+^ CD4 T cells and their expression of KLRG1, alongside **(H)** their production of IL-10 following PMA/I stimulation. **(I)** Lung neutrophil (Ly6G^+^CD11b^+^CD90^-^CD19^-^Autofluorescence^-^) numbers. Data is n = 8 mice per group pooled from 2 independent experiments.
(PDF)

S5 Fig IL-6 promotes IL-27 after RSV infection. 8 week old BALB/c female mice were infected with 8 x 10^5^ ffu of RSV A2 and dosed with either αIL-6 or isotype control antibody i.p. between days -1 and 3 p.i. (A) Gating strategy for myeloid cells in the lungs, plots represent day 1 p.i.. (B) Representative histograms of IL-27^+^, IL-6^+^ and TNF^+^ alveolar macrophages in the BAL, and (C) IL-27^+^ neutrophils, Ly6C^+^ monocytes, CD11b^+^ and CD11b^-^ DCs in the lungs. Gating is shown and dotted lines represent the median fluorescent intensity of cells from uninfected mice. Data is representative of n = 5 mice per group per time points, from 2 independent repeats.
(PDF)

S6 Fig IL-6 does not regulate myeloid cell numbers after RSV infection. 8 week old BALB/c mice were infected with 8 x 10^5^ ffu of RSV A2 i.n. and given 0.5 mg of either HRPN (IgG_1_) or MP5-20F3 (αIL-6) i.p. on day -1 p.i. and 0.25 mg i.p. every other day after that. (A) Lung cells were incubated with brefeldin A for 6 hrs and the frequency of IL-6^+^, IL-27^+^ and TNF^+^ lung alveolar macrophages (AF^+^CD68^+^CD11c^+^) was determined by flow cytometry. (B) The number of alveolar macrophages, neutrophils, monocyte/macrophages, CD11b^+^ and CD11b^-^ DCs was determined by flow cytometry. (C) MHCII upregulation on BAL alveolar macrophages was determined at day 4 p.i.. (D) MHCII expression by IL-27^+^ versus total alveolar macrophages at day 4 p.i. Data is n = 5 mice per group per timepoint and representative of 2 independent experiments.
(PDF)

S7 Fig KLRG1 identifies a highly activated subset of Tregs. 8 week old BALB/c female mice were infected with 8 x 10^5^ ffu of RSV A2 and sacrificed at day 4 p.i. **(A)** Foxp3 and CD4 staining in BAL, Lung and lung draining lymph nodes were analysed. **(B)** All Foxp3^+^ (grey filled histograms) and KLRG1^+^ Foxp3^+^ Tregs (colour filled histograms) were analyzed for their expression of key markers. All CD45^+^ cells (black line) are shown as a control. **(C)** Mice were treated as in Figs 7 and 8, and at day 10 p.i. lung cells were analyzed for the number of Tregs and proportion expression KLRG1 and Helios. Data represents n = 5 mice.
(PDF)
