# Supplementary material for: Early IL-6 signalling promotes IL-27 dependent maturation of regulatory T cells in the lungs and resolution of viral immunopathology
Source: PLoS Pathog. 2017 Sep 27;13(9):e1006640. doi: 10.1371/journal.ppat.1006640 (PMC5633202; doi:10.1371/journal.ppat.1006640)
Supplement: S1 Fig — 8 week old BALB/c mice were infected with 8 x 10^5^ ffu of RSV A2 i.n.. Mice were treated with anti-IL6 or IgG1 isotype control i.p. from days -1 to 13 p.i. IL-6 was measured by ELISA in the BAL, lung homogenate and serum at the indicated days post infection. Data is representative of n = 2 independent repeats of n = 5 mice per time point. Kruskal-Wallis H test was carried out between baseline and each d.p.i. (PDF) [file ppat.1006640.s001.pdf]

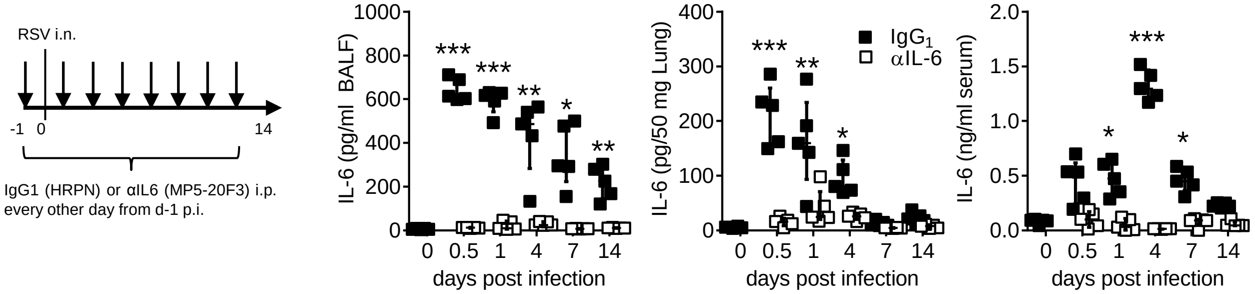

Supplemental figure 1. Anti-IL-6 treatment depletes RSV induced IL-6 both locally and systemically. 8 week old BALB/c mice were infected with 8 x 10^5 ffu of RSV A2 i.n.. Mice were treated with anti-IL6 or IgG_1 isotype control i.p. from days -1 to 13 p.i. IL-6 was measured by ELISA in the BAL**,** lung homogenate and serum at the indicated days post infection. Data is representative of n = 2 independent repeats of n = 5 mice per time point. Kruskal-Wallis H test was carried out between baseline and each d.p.i.
