# Supplementary material for: Early IL-6 signalling promotes IL-27 dependent maturation of regulatory T cells in the lungs and resolution of viral immunopathology
Source: PLoS Pathog. 2017 Sep 27;13(9):e1006640. doi: 10.1371/journal.ppat.1006640 (PMC5633202; doi:10.1371/journal.ppat.1006640)
Supplement: S2 Fig — 8 week old BALB/c mice were infected with 8 x 10^5^ ffu of RSV A2 i.n. and given 0.5 mg of either HRPN (IgG1) or MP5-20F3 (αIL-6) i.p. on day -1 p.i. and 0.25 mg i.p. every other day after that. Mice were euthanized at days 4, 7 and 14 p.i. Flow cytometry was used to determine the proportion of KbM282-90+ CD8+ T cells in the lungs (A) and lymph node (B). Data is representative of 5 mice per group and 2 independent repeats. Plots depict the median percentage tetramer positive cells within the total lymphocyte population for each group at each time point. (PDF) [file ppat.1006640.s002.pdf]

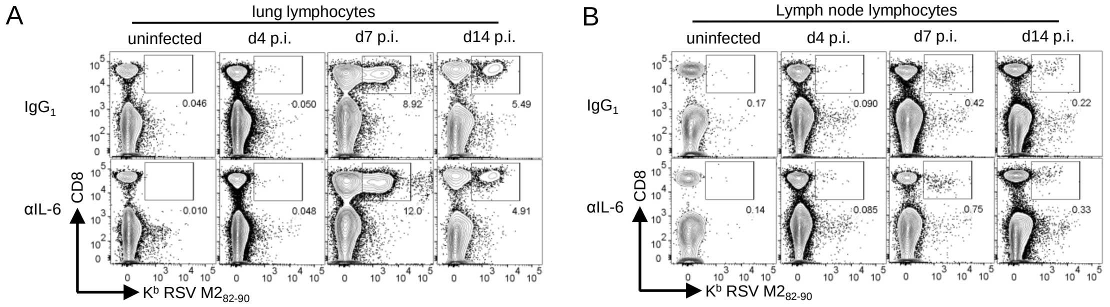

Supplemental Figure 2. IL-6 regulates the proportion of virus specific CD8 T cells. 8 week old BALB/c mice were infected with 8 x 10^5 ffu of RSV A2 i.n. and given 0.5 mg of either HRPN (IgG$_1$) or MP5-20F3 (αIL-6) i.p. on day -1 p.i. and 0.25 mg i.p. every other day after that. Mice were euthanized at days 4, 7 and 14 p.i. Flow cytometry was used to determine the proportion of K^{b}M2$_{82\text{-}90}$$^+$ CD8$^+$ T cells in the lungs **(A)** and lymph node **(B)**. Data is representative of 5 mice per group and 2 independent repeats. Plots depict the median percentage tetramer positive cells within the total lymphocyte population for each group at each time point.
