# Supplementary material for: Early IL-6 signalling promotes IL-27 dependent maturation of regulatory T cells in the lungs and resolution of viral immunopathology
Source: PLoS Pathog. 2017 Sep 27;13(9):e1006640. doi: 10.1371/journal.ppat.1006640 (PMC5633202; doi:10.1371/journal.ppat.1006640)
Supplement: S3 Fig — 8 week old BALB/c female mice were infected with 8 x 10^5^ ffu of RSV A2 i.n. and dosed with either αIL-6 or isotype control antibody as shown in Fig 5A. Clinical symptom scores were taken daily. Data are representative of n = 5 mice per group and 2 independent experiments. Area under the curve (AUC) was calculated and Mann-Whitney test between control and αIL-6 treated groups for each regime carried out. (PDF) [file ppat.1006640.s003.pdf]

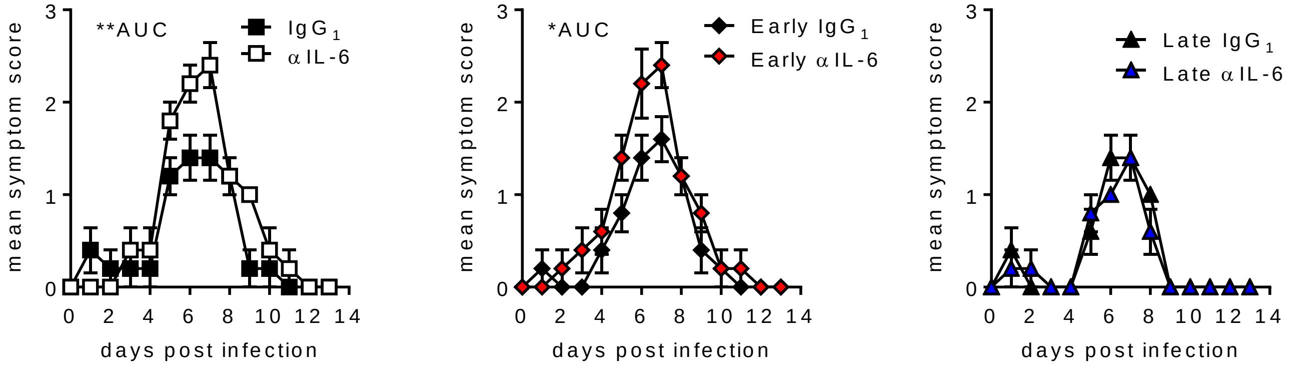

Supplemental Figure 3. Early, but not late, IL-6 signalling regulates RSV induced disease. 8 week old BALB/c female mice were infected with 8 x 10^5 ffu of RSV A2 i.n. and dosed with either αIL-6 or isotype control antibody as shown in Figure 5A. Clinical symptom scores were taken daily. Data are representative of n = 5 mice per group and 2 independent experiments. Area under the curve (AUC) was calculated and Mann-Whitney test between control and αIL-6 treated groups for each regime carried out.
