# Supplementary material for: Early IL-6 signalling promotes IL-27 dependent maturation of regulatory T cells in the lungs and resolution of viral immunopathology
Source: PLoS Pathog. 2017 Sep 27;13(9):e1006640. doi: 10.1371/journal.ppat.1006640 (PMC5633202; doi:10.1371/journal.ppat.1006640)
Supplement: S4 Fig — 8 week old BALB/c female mice were infected with 40 pfu of IAV PR8 and dosed with either αIL-6 or isotype control antibody i.p. between days -1 and 3 p.i. (A) Weight loss was monitored daily, area under the curve (AUC) was used to test statistical significance. (B-H) Mice were euthanized at day 10 p.i. and (B) IL-6, IL-10 and IL-27 in the BAL and (C) IFN-γ in the lungs were measured by ELISA. (D) The frequency of antigen experienced CD8+ T cells (PD1+CD44+CD62L-) and CD4+ T cells in the lungs. (E) The frequency of lung IFN-γ+ CD4 T cells in the lungs, and (F) the proportion that were IL-10+ after PMA/I stimulation. (G) Foxp3+ CD4 T cells and their expression of KLRG1, alongside (H) their production of IL-10 following PMA/I stimulation. (I) Lung neutrophil (Ly6G+CD11b+CD90-CD19-Autofluorescence-) numbers. Data is n = 8 mice per group pooled from 2 independent experiments. (PDF) [file ppat.1006640.s004.pdf]

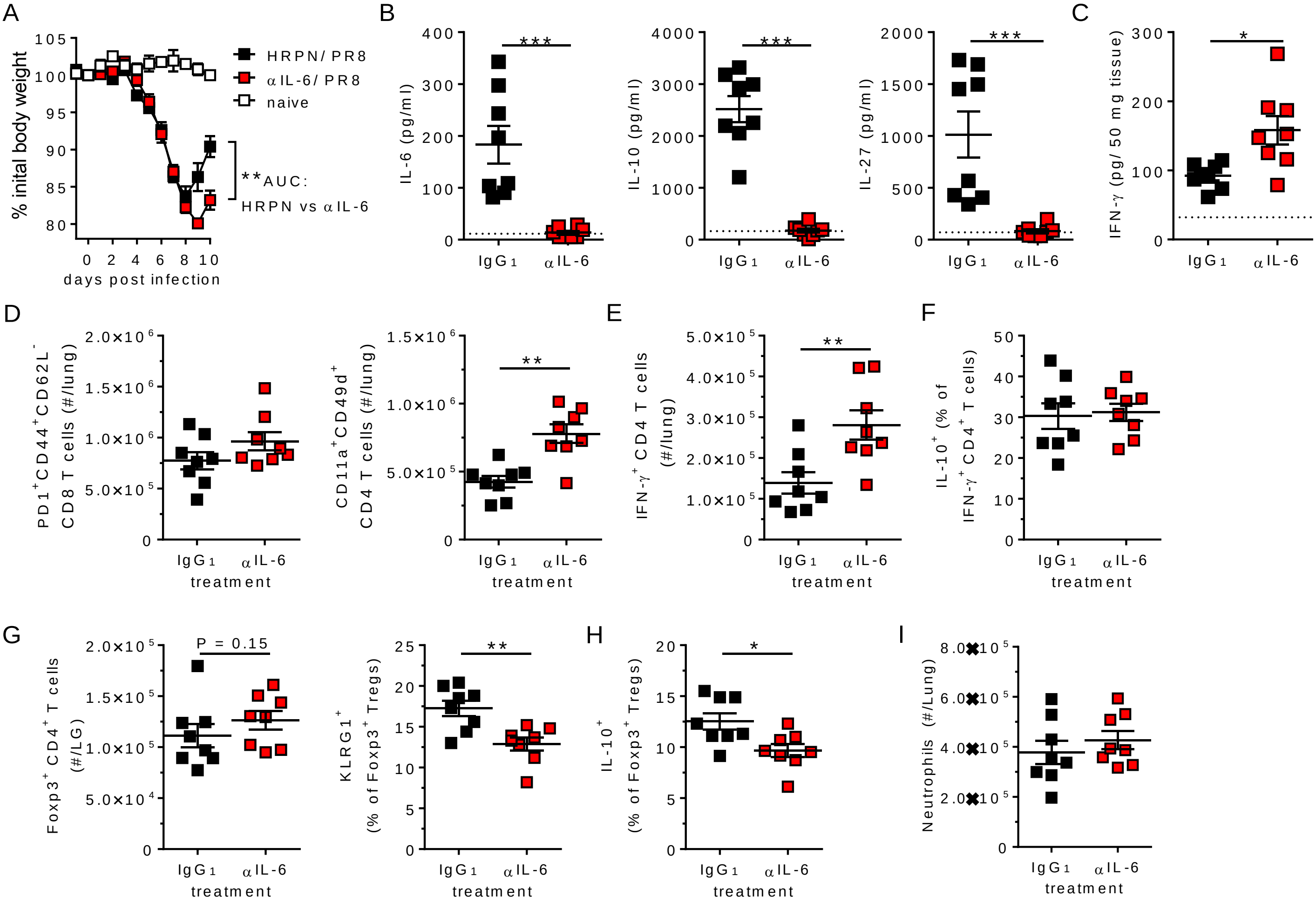

Supplemental Figure 4. IL-6 regulates disease resolution after influenza A virus infection. 8 week old BALB/c female mice were infected with 40 pfu of IAV PR8 and dosed with either αIL-6 or isotype control antibody i.p. between days -1 and 3 p.i. (A) Weight loss was monitored daily, area under the curve (AUC) was used to test statistical significance. (B-H) Mice were euthanized at day 10 p.i. and (B) IL-6, IL-10 and IL-27 in the BAL and (C) IFN-γ in the lungs were measured by ELISA. (D) The frequency of antigen experienced CD8$^+$ T cells (PD1$^+$CD44$^+$CD62L$^-$) and CD4$^+$ T cells in the lungs. (E) The frequency of lung IFN-γ$^+$ CD4 T cells in the lungs, and (F) the proportion that were IL-10$^+$ after PMA/I stimulation. (G) Foxp3$^+$ CD4 T cells and their expression of KLRG1, alongside (H) their production of IL-10 following PMA/I stimulation. (I) Lung neutrophil (Ly6G$^+$CD11b$^+$CD90$^-$CD19$^-$Autofluorescence$^-$) numbers. Data is n = 8 mice per group pooled from 2 independent experiments.
