# Supplementary material for: Early IL-6 signalling promotes IL-27 dependent maturation of regulatory T cells in the lungs and resolution of viral immunopathology
Source: PLoS Pathog. 2017 Sep 27;13(9):e1006640. doi: 10.1371/journal.ppat.1006640 (PMC5633202; doi:10.1371/journal.ppat.1006640)
Supplement: S5 Fig — 8 week old BALB/c female mice were infected with 8 x 10^5^ ffu of RSV A2 and dosed with either αIL-6 or isotype control antibody i.p. between days -1 and 3 p.i. (A) Gating strategy for myeloid cells in the lungs, plots represent day 1 p.i.. (B) Representative histograms of IL-27+, IL-6+ and TNF+ alveolar macrophages in the BAL, and (C) IL-27+ neutrophils, Ly6C+ monocytes, CD11b+ and CD11b- DCs in the lungs. Gating is shown and dotted lines represent the median fluorescent intensity of cells from uninfected mice. Data is representative of n = 5 mice per group per time points, from 2 independent repeats. (PDF) [file ppat.1006640.s005.pdf]

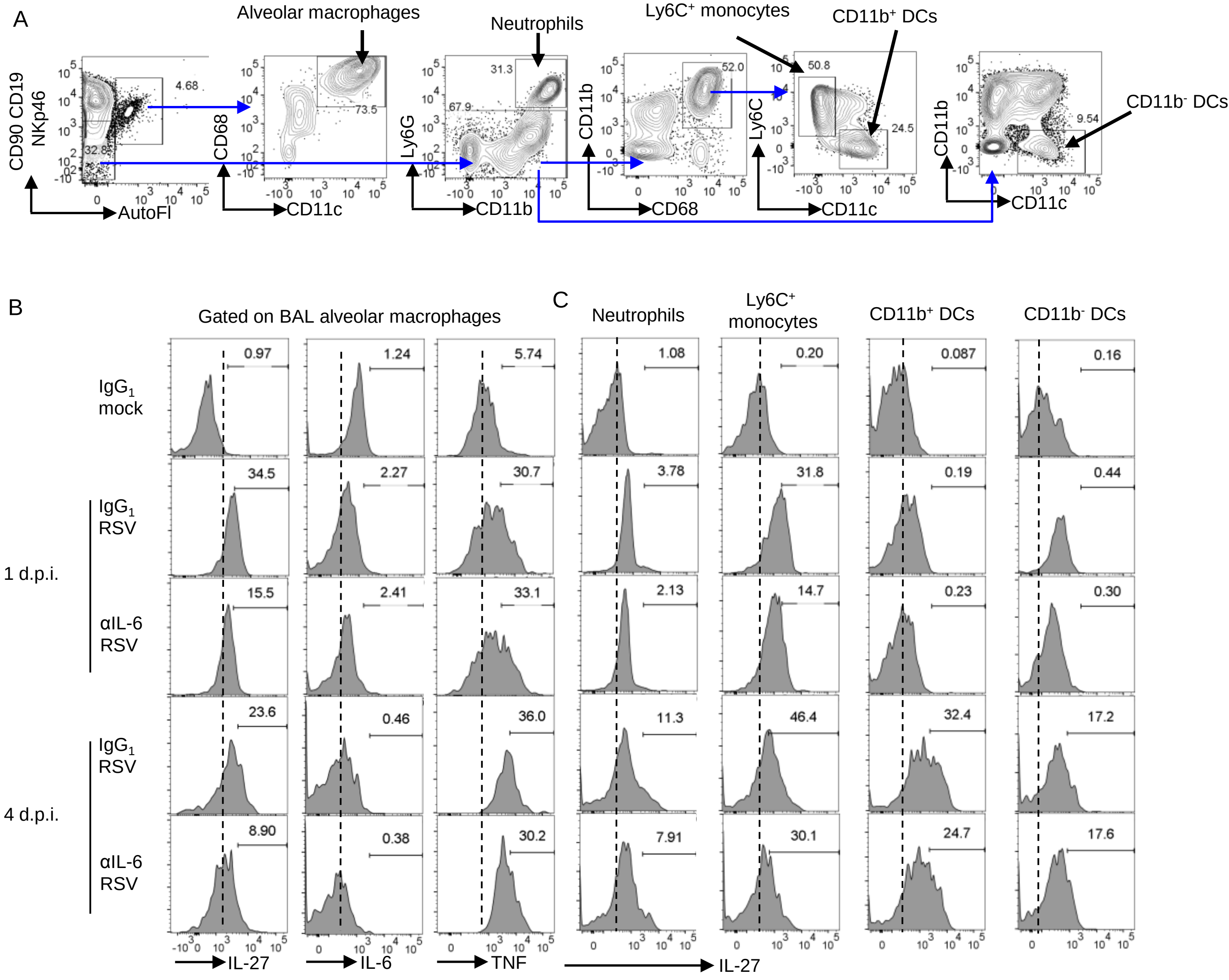

Supplemental Figure 5. IL-6 promotes IL-27 after RSV infection. 8 week old BALB/c female mice were infected with 8 x 10^5 ffu of RSV A2 and dosed with either αIL-6 or isotype control antibody i.p. between days -1 and 3 p.i. (A) Gating strategy for myeloid cells in the lungs, plots represent day 1 p.i.. (B) Representative histograms of IL-27$^+$, IL-6$^+$ and TNF$^+$ alveolar macrophages in the BAL, and (C) IL-27$^+$ neutrophils, Ly6C$^+$ monocytes, CD11b$^+$ and CD11b$^-$ DCs in the lungs. Gating is shown and dotted lines represent the median fluorescent intensity of cells from uninfected mice. Data is representative of n = 5 mice per group per time points, from 2 independent repeats.
