# Supplementary material for: Early IL-6 signalling promotes IL-27 dependent maturation of regulatory T cells in the lungs and resolution of viral immunopathology
Source: PLoS Pathog. 2017 Sep 27;13(9):e1006640. doi: 10.1371/journal.ppat.1006640 (PMC5633202; doi:10.1371/journal.ppat.1006640)
Supplement: S6 Fig — 8 week old BALB/c mice were infected with 8 x 10^5^ ffu of RSV A2 i.n. and given 0.5 mg of either HRPN (IgG1) or MP5-20F3 (αIL-6) i.p. on day -1 p.i. and 0.25 mg i.p. every other day after that. (A) Lung cells were incubated with brefeldin A for 6 hrs and the frequency of IL-6+, IL-27+ and TNF+ lung alveolar macrophages (AF+CD68+CD11c+) was determined by flow cytometry. (B) The number of alveolar macrophages, neutrophils, monocyte/macrophages, CD11b+ and CD11b- DCs was determined by flow cytometry. (C) MHCII upregulation on BAL alveolar macrophages was determined at day 4 p.i.. (D) MHCII expression by IL-27+ versus total alveolar macrophages at day 4 p.i. Data is n = 5 mice per group per timepoint and representative of 2 independent experiments. (PDF) [file ppat.1006640.s006.pdf]

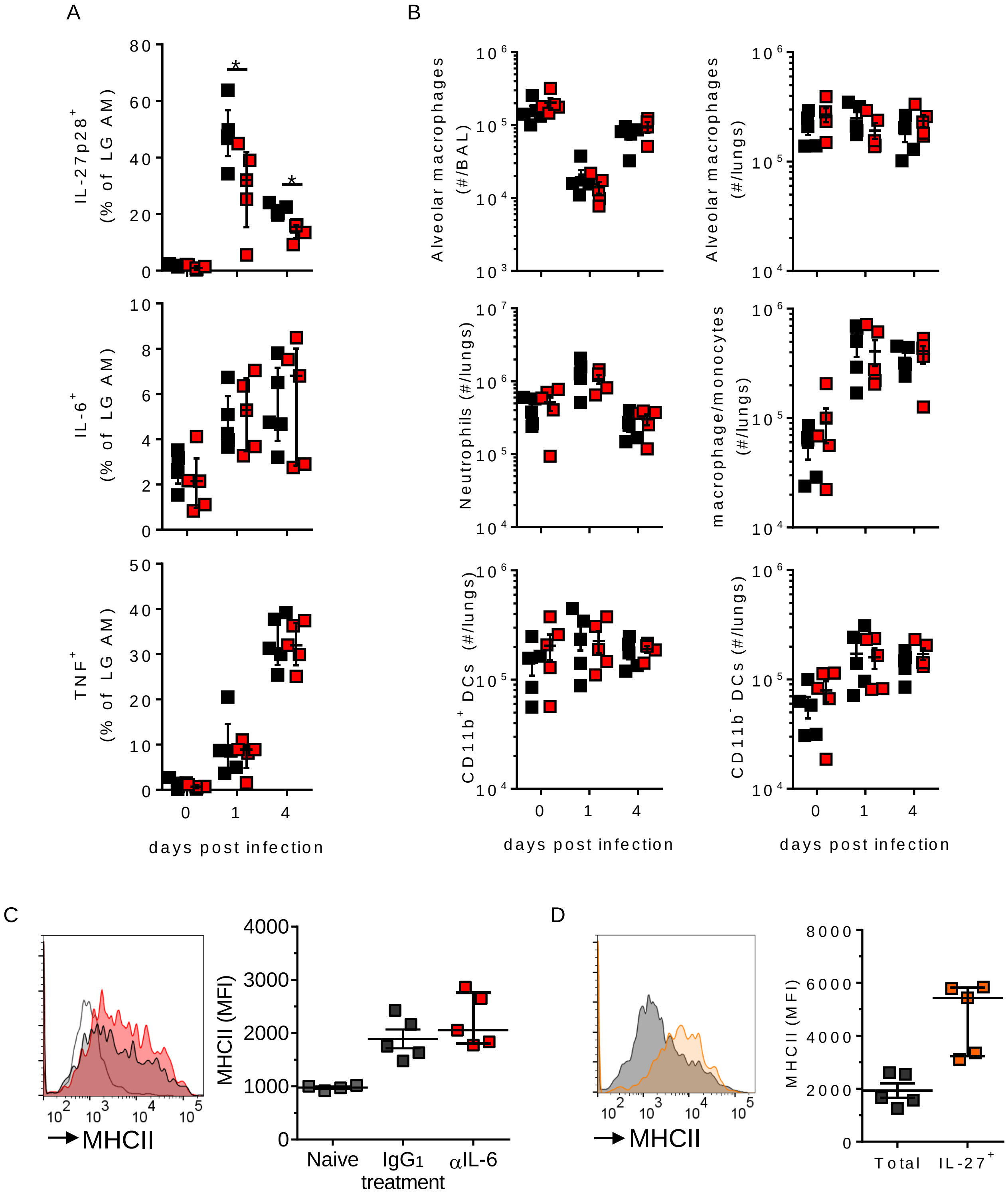

Supplemental Figure 6. IL-6 does not regulate myeloid cell numbers after RSV infection. 8 week old BALB/c mice were infected with 8 x 10^5 ffu of RSV A2 i.n. and given 0.5 mg of either HRPN (IgG_1) or MP5-20F3 (αIL-6) i.p. on day -1 p.i. and 0.25 mg i.p. every other day after that. (A) Lung cells were incubated with brefeldin A for 6 hrs and the frequency of IL-6$^+$, IL-27$^+$ and TNF$^+$ lung alveolar macrophages (AF$^+$CD68$^+$CD11c$^+$) was determined by flow cytometry. (B) The number of alveolar macrophages, neutrophils, monocyte/macrophages, CD11b$^+$ and CD11b$^-$ DCs was determined by flow cytometry. (C) MHCII upregulation on BAL alveolar macrophages was determined at day 4 p.i.. (D) MHCII expression by IL-27$^+$ versus total alveolar macrophages at day 4 p.i. Data is n = 5 mice per group per timepoint and representative of 2 independent experiments.
