# Supplementary material for: Early IL-6 signalling promotes IL-27 dependent maturation of regulatory T cells in the lungs and resolution of viral immunopathology
Source: PLoS Pathog. 2017 Sep 27;13(9):e1006640. doi: 10.1371/journal.ppat.1006640 (PMC5633202; doi:10.1371/journal.ppat.1006640)
Supplement: S7 Fig — 8 week old BALB/c female mice were infected with 8 x 10^5^ ffu of RSV A2 and sacrificed at day 4 p.i. (A) Foxp3 and CD4 staining in BAL, Lung and lung draining lymph nodes were analysed. (B) All Foxp3+ (grey filled histograms) and KLRG1+ Foxp3+ Tregs (colour filled histograms) were analyzed for their expression of key markers. All CD45+ cells (black line) are shown as a control. (C) Mice were treated as in Figs 7 and 8, and at day 10 p.i. lung cells were analyzed for the number of Tregs and proportion expression KLRG1 and Helios. Data represents n = 5 mice. (PDF) [file ppat.1006640.s007.pdf]

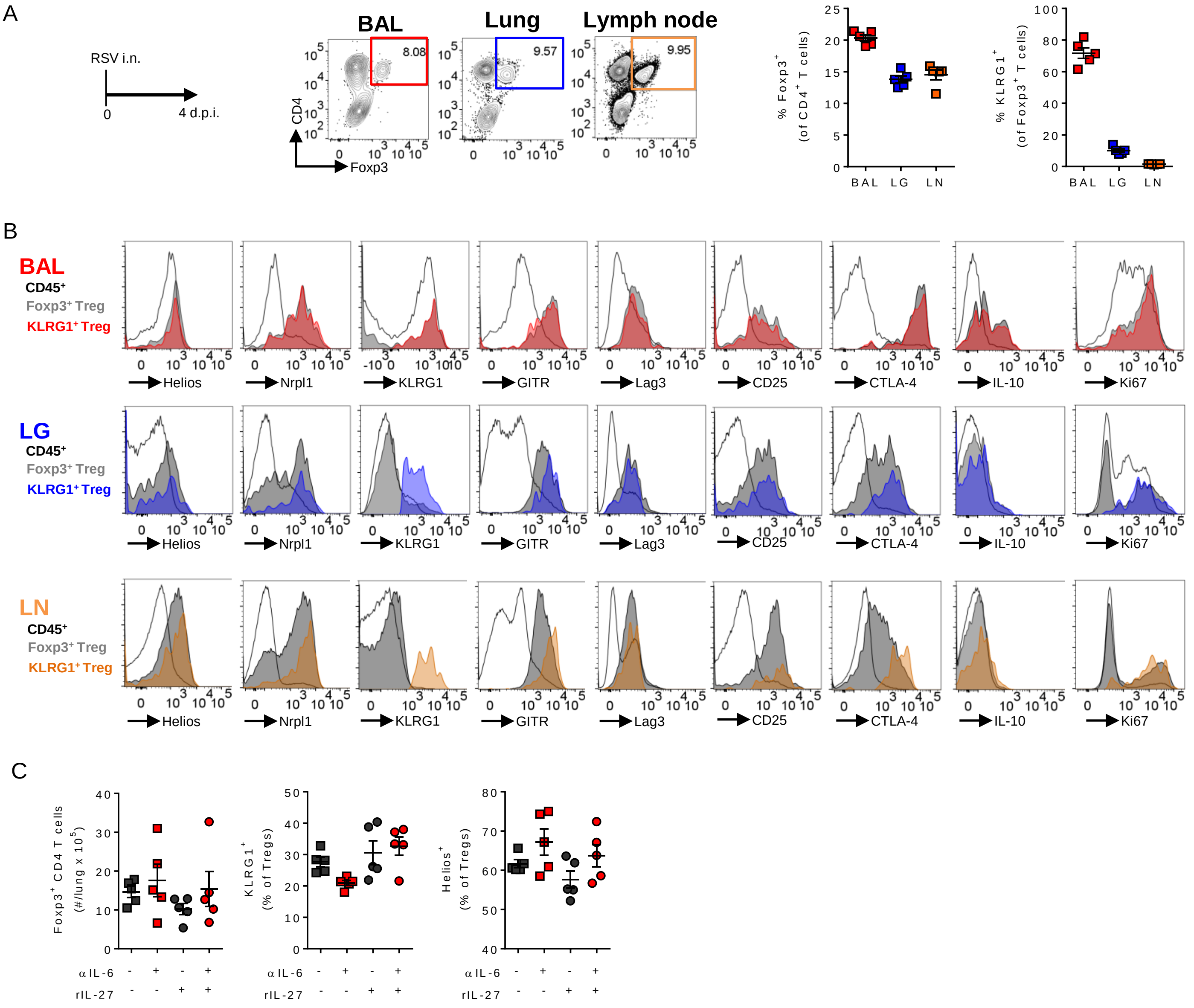

Supplemental Figure 7. KLRG1 identifies a highly activated subset of Tregs. 8 week old BALB/c female mice were infected with 8 x 10^5 ffu of RSV A2 and sacrificed at day 4 p.i. (A) Foxp3 and CD4 staining in BAL, Lung and lung draining lymph nodes were analysed. (B) All Foxp3$^+$ (grey filled histograms) and KLRG1$^+$ Foxp3$^+$ Tregs (colour filled histograms) were analyzed for their expression of key markers. All CD45$^+$ cells (black line) are shown as a control. (C) Mice were treated as in Fig 7 and 8, and at day 10 p.i. lung cells were analyzed for the number of Tregs and proportion expression KLRG1 and Helios. All CD45$^+$ cells (black line) are shown as a control. Data represents n = 5 mice.
